# Analogs of a Natural Peptaibol Exert Anticancer Activity in Both Cisplatin- and Doxorubicin-Resistant Cells and in Multicellular Tumor Spheroids

**DOI:** 10.3390/ijms22168362

**Published:** 2021-08-04

**Authors:** Naike Casagrande, Cinzia Borghese, Laura Gabbatore, Laura Morbiato, Marta De Zotti, Donatella Aldinucci

**Affiliations:** 1 Molecular Oncology, Centro di Riferimento Oncologico di Aviano (CRO) IRCCS, 33081 Aviano, Italy; naike.casagrande@cro.it (N.C.); cpborghese@cro.it (C.B.); 2 Department of Chemistry, University of Padova, 35131 Padova, Italy; laura.gabbatore@unipd.it (L.G.); laura.morbiato.1@unipd.it (L.M.)

**Keywords:** anticancer peptide, alpha-helix, drug-resistant tumors, Hodgkin lymphoma, ovarian cancer, multicellular tumor spheroids, circular dichroism, peptide-membrane interaction

## Abstract

Peptaibols, by disturbing the permeability of phospholipid membranes, can overcome anticancer drug resistance, but their natural hydrophobicity hampers their administration. By a green peptide synthesis protocol, we produced two water-soluble analogs of the peptaibol trichogin GA IV, termed K6-Lol and K6-NH2. To reduce production costs, we successfully explored the possibility of changing the naturally occurring 1,2-aminoalcohol leucinol to a C-terminal amide. Peptaibol activity was evaluated in ovarian cancer (OvCa) and Hodgkin lymphoma (HL) cell lines. Peptaibols exerted comparable cytotoxic effects in cancer cell lines that were sensitive—and had acquired resistance—to cisplatin and doxorubicin, as well as in the extrinsic-drug-resistant OvCa 3-dimensional spheroids. Peptaibols, rapidly taken up by tumor cells, deeply penetrated and killed OvCa-spheroids. They led to cell membrane permeabilization and phosphatidylserine exposure and were taken up faster by cancer cells than normal cells. They were resistant to proteolysis and maintained a stable helical structure in the presence of cancer cells. In conclusion, these promising results strongly point out the need for further preclinical evaluation of our peptaibols as new anticancer agents.

## 1. Introduction

In the last two decades, the cumulative number of peptides approved in major pharmaceutical markets has doubled [1]. Peptide active agents overcome the known disadvantages of both small molecules and proteins. They have target specificity, rarely show off-target toxicity, are far cheaper than biological molecules (therapeutic proteins), have better tissue penetration ability and reduced immunogenicity than antibodies [2]. In this context, naturally occurring, bioactive peptides are of course the first choice for the development of active agents. The major drawbacks hampering their use are their low oral bioavailability and plasma stability. To improve peptide stability, researchers devised a variety of tricks, from the synthesis of stapled peptides [3], a gold mine for drug discovery strategies, to the development of cyclic peptides [4], and to the exploitation of peptide-decorated nanoparticles [5]. On the other hand, nature offers a family of intrinsically stable peptides called peptaibols, non-ribosomally synthesized by fungi as part of their defense mechanism against other microorganisms. Peptides belonging to that family possess, even when short, exceptionally stable helical 3D structures. This feature stems from the presence of several residues of the sterically hindered, helix-inducer, natural amino acid α-aminoisobutyric acid (Aib) in their sequence.

Peptaibols are particularly promising as anticancer drugs because they act by perturbing the permeability of phospholipid membranes, a mechanism less likely to generate drug resistance [6,7,8]. On the other hand, they are naturally hydrophobic peptides and this hampers their efficient delivery to their biological target. We recently found that water-soluble peptaibol analogs, featuring Gly-to-Lys substitutions, possess anticancer activity [6]. Besides, through the application of a biorational approach, we recently designed water-soluble peptaibol analogs with improved bioactivity against fungal plant pathogens [9].

The synthesis of peptaibols is not trivial, as the steric hindrance of Aib makes it poorly reactive. Nonetheless, we have recently proposed an effective, ecofriendly strategy [9] that we are also applying here. Such a procedure for the synthesis of peptaibols is also less expensive than the standard protocol, and represents a step forward towards the translation of peptaibols to the market; however, it did not solve the main problem connected with the production of peptaibols: the presence of a C-terminal aminoalcohol (featured in the natural sequences), that forces the use of costly, preloaded resins for their production by solid-phase peptide synthesis [10].

We herein propose the substitution of the naturally occurring C-terminal Leucinol for the far cheaper Leucine amide and test if this modification affects the bioactivity of the peptide analogs. To this end, we synthesized two water-soluble analogs of the short-length peptaibol trichogin GA IV, termed K6-Lol and K6-NH2.

To study the new peptaibols activity we used tumor models derived from ovarian cancer (OvCa) and Hodgkin lymphoma (HL), including for the former cisplatin-resistant cell lines, and for the latter doxorubicin-resistant cells [11].

Epithelial OvCa is the ninth most prevalent cancer and the fifth most common cause of cancer-related death in women [12]. A characteristic of OvCa is the accumulation of fluid in the peritoneal cavity, the malignant ascites [13]. Ascites contain aggregates of tumor cells called multicellular spheroids (MCTSs), resistant to anticancer drugs and capable of disseminating in the peritoneum and forming metastasis [13,14]. Most OvCa patients are responsive to chemotherapy at first but then intrinsic and acquired resistance to cisplatin [15] causes tumor relapse [16].

HL is a B-cell lymphoid malignancy. The first-line therapy is the ABVD (Adriamycin, Bleomycin, Vinblastine, and Dacarbazine) regimen, which includes Adriamycin (doxorubicin), an antineoplastic agent, whose efficacy is limited by cardiotoxicity and the development of tumor resistance [17]. Although chemotherapy has a high response rate, 20–30% of cHL patients will experience relapse [18].

Our hypothesis is that peptaibols, by interacting and perturbing the cellular membrane of cancer cells [6,19], could exert antitumoral activity in both cisplatin- and doxorubicin-resistant cells, as well as in OvCa derived MCTSs.

We herein describe the synthesis (by green synthesis) of the water-soluble peptaibol analogs K6-Lol and K6-NH2, and determine/compare (i) their proteolytic and plasma stability, (ii) their mechanism of action and (iii) cytotoxicity in cisplatin and doxorubicin-resistant cancer cells from HL lymphoma (Hodgkin Reed Sternberg, HRS) and from OvCa.

## 2. Results and Discussion

### 2.1. Peptide Synthesis

**Scheme 1:**
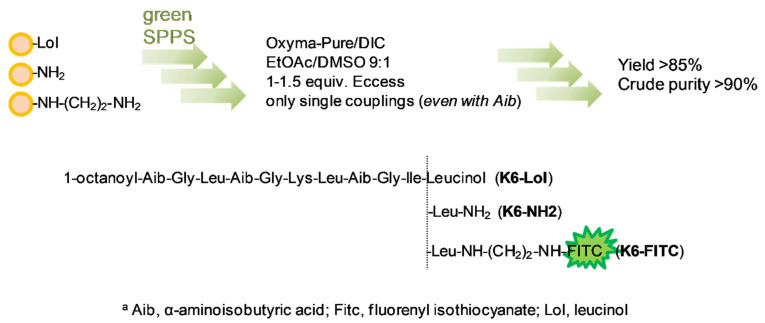
Synthesis and chemical structure of K6-Lol, K6-NH2 and K6-FITC^a^.

**Figure 1:**
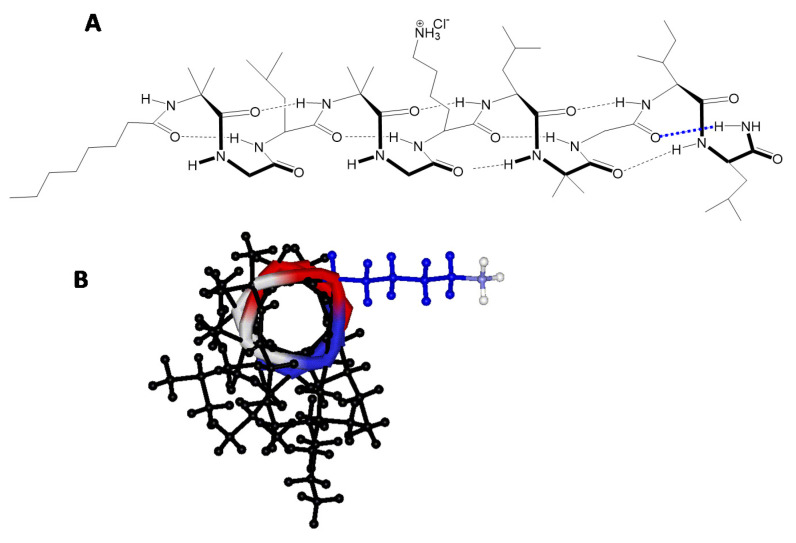
K6-NH2 structure. (**A**) The helical structure of K6-NH2 is strengthened by the presence of an additional H-bond, highlighted in blue. (**B**) Three-dimensional structural model for K6-NH2. The natural—albeit weak—amphiphilic structure of native peptaibol is enhanced by the presence of a Lys residue on the hydrophilic face (Lys side chain is highlighted in blue).

The synthesis of K6-Lol, K6-NH2 and K6-FITC (used for internalization studies) was carried out by means of a green protocol, using Oxyma-pure and diisopropylcarbodiimide (DIC), classified as two of the greenest coupling reagents by the ACS Green Chemistry Institute^®^ Pharmaceutical Roundtable [20], and a mixture of ethyl acetate and dimethylsulfoxide 9:1 as solvent (Scheme 1). Purification was successfully achieved by medium-pressure chromatography (Isolera Prime, Biotage) using mostly water and less than 500 mL of acetonitrile (all purifications included). The yields after purification were always more than 70%, although the last step of K6FITC production was complicated by solubility issues of the fully protected peptide precursor. The obtained peptide purity was >96%. Characterizations are reported in the Supporting Information. The representative helical structure of K6-NH2 is shown in Figure 1.

### 2.2. Peptaibols Are Stable in Serum and Resistant to Enzymatic Digestion

K6-Lol and K6-NH2, tested in human serum, were found to be completely stable for more than 24 h (Supplemental Figure S1). No changes in the HPLC profile were detected. We performed a stability assay also in the presence of single proteolytic enzymes. Although the presence of a Lys residue should make them sensitive to the action of trypsin, K6-NH2 was fully stable for more than 24 h in the presence of that enzyme as no changes in its HPLC profile were detected (Supplemental Figures S2 and S3). The peptaibol was also found to be fully stable in contact with Pronase for more than 24 h (Supplemental Figures S3 and S4).

### 2.3. In Vitro Cytotoxicity of Peptaibols in cHL and OvCa Cells

Several antimicrobial peptides (AMPs) and peptaibols [6] have shown antitumor activity, and some AMPs have even entered the clinical practice [21]; thus, we evaluated the potential anticancer activity of K6-Lol and of the less expensive analog K6-NH2. Peptaibol activity was evaluated in two different cancer models: ovarian cancer (OvCa) (solid tumor) and Hodgkin lymphoma (HL) (hematological malignancy). We used a panel of HL cell lines (L-1236, L-428, HDLM-2, and KM-H2) and OvCa cell lines (OVCAR5, SKOV3, and A2780). The main obstacles for successful chemotherapy are represented by drug toxicity and drug resistance [22]. Cisplatin- and doxorubicin-resistance is a frequent cause of treatment failure in OvCa and cHL [23,24,25]. Therefore, we also evaluated peptaibols activity in A2780cis, the cisplatin-resistant clone of A2780 cells (Table 1, Supplemental Figure S5A), and in KM-H2dx and HDLM-2dx, the doxorubicin-resistant clones of KM-H2 and HDLM-2 cell lines, respectively (Table 1, Supplemental Figure S5B,C). A2780cis cells have a cross-drug resistance to doxorubicin (Table 1, Supplemental Figure S5A) and both KM-H2dx and HDLM-2dx are less sensitive to cisplatin than their parental clones (KM-H2 and HDLM-2) (Table 1, Supplemental Figure S5B,C). Moreover, HDLM-2 cells are intrinsically resistant to Brentuximab vedotin (BV), an antibody–drug conjugate used in relapsed/refractory HL patients [17,26]. We found that both peptaibols decreased tumor growth of cHL and OvCa cell lines in a dose dependent manner, with IC_50_ ranging from 7.2 to 13.2 µM in cHL cells and from 7.5 to 10.5 µM in OvCa cells (Table 1).

**Figure 2:**
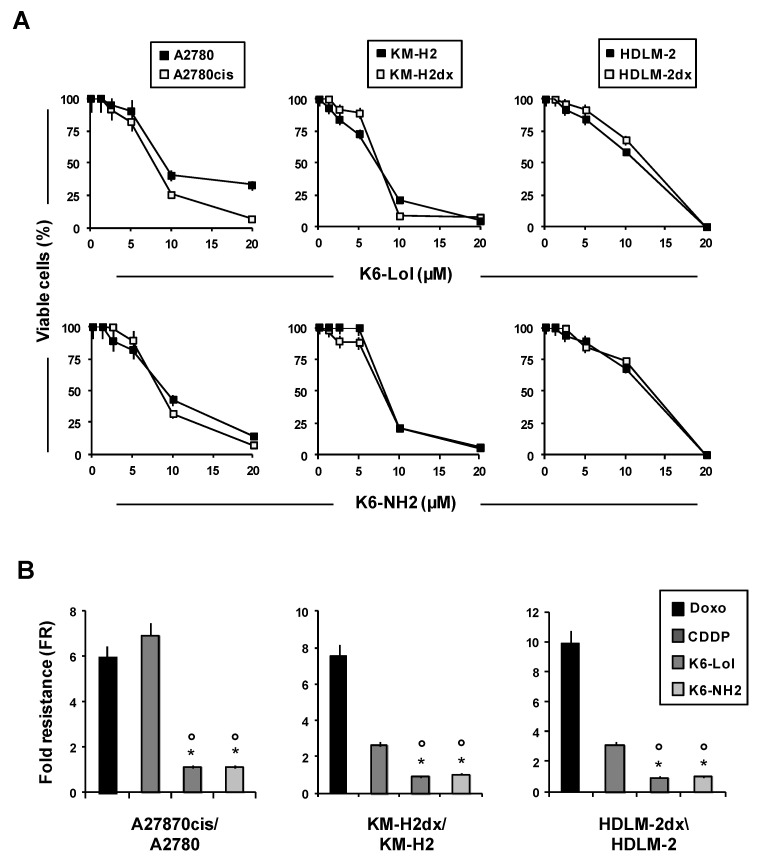
K6-Lol and K6-NH2 kill HRS and OvCa cells, and are equally active in doxorubicin and cisplatin resistant cells. Cytotoxicity assay. Tumor cells were exposed to increasing concentrations of peptaibols. (**A**) After 24 h cell viability was evaluated with MTT assay in A2780 and A2780cis, and MTS assay in KM-H2, KM-H2dx, HDLM-2 and HDLM-2dx. Results are mean and SD of three independent experiments. (**B**) Fold resistance, calculated as the ratio of the IC_50_ of drug-resistant cells to that of their corresponding parental cell lines. * *p* < 0.05 vs. doxorubicin and ° *p* < 0.05 vs. cisplatin. Student’s *t* test. Doxo, doxorubicin; CDDP, cisplatin.

Multidrug resistance (MDR) proteins are integral membrane proteins that extrude cytotoxic drugs from living cells, including doxorubicin and cisplatin [27]. It is of note that MDR1/ABCB1/P-glycoprotein is up-regulated in BV-resistant HRS cells [26]. Intrinsic or acquired MDR is one of the main reasons for chemotherapy failure and several MDR reversal agents were developed and entered into clinical trials [26,28]. As MDR inhibitors often failed due to toxicity or the occurrence of resistance, finding novel agents that overcome MDR activity is a clinical challenge [27]. AMPs were found to overcome drug resistance in OvCa cells [29] and in ABCB1-overexpressing epidermoid carcinoma cells [30]. Thus, we evaluated the activity of our peptaibols in cell lines with acquired resistance to doxorubicin (HDLM-2dx and KMH2-dx) and cisplatin (A2780cis). We found that peptaibols exerted a comparable activity in both drug-sensitive cell lines (A2780, HDLM-2, and KM-H2) and their corresponding drug-resistant clones (A2780cis, HDLM-2dx, and KM-H2dx) (Figure 2A and Table 1).

For each cell line, we calculated the IC_50_, i.e., the concentration of drug to decrease cell viability to 50%. The ratio of the IC_50_ of the drug-resistant cell line to that of its parental cell line is the fold resistance (FR). The higher the FR value, the higher the drug-resistance. HDLM-2dx and KM-H2dx were both about nine times less sensitive to doxorubicin than their parental cell lines (FR = 9), and 3.11 and 2.6 to cisplatin (FR = 3.11 and FR = 2.6, respectively), but had comparable sensitivity to K6-Lol and K6-NH2 with FR values near to 1 (Figure 2B). In addition, our results suggest that peptaibols may be active in HRS cells with intrinsic resistance to BV (HDLM-2 cells) [31,32], or likely with acquired resistance to BV, characterized by the up-regulation of MDR1 (L-428R and KM-H2R) [26].

A2780cis were more resistant to both cisplatin (FR = 8) and doxorubicin (FR = 5.6) than parental A2780 cells but had similar sensitivity to both peptaibols (FR value near 1.0) (Figure 2B). These results demonstrated that the substitution of Lol with Leu-NH2 (leading to the less expensive analog K6-NH2) did not significantly modify the cytotoxic effects of peptaibols. Both peptaibols were almost inactive until a minimum concentration was reached (see dose–response curves in Figure 2A, showing a sharp decline of the kill-curve after reaching a threshold concentration), which is consistent with the fact that peptaibols determine membrane lysis and trigger citotoxicity only when a critical peptide concentration on the surface membrane is reached [6].

### 2.4. Peptaibols Deeply Penetrate and Kill SKOV3-MCTSs

**Figure 3:**
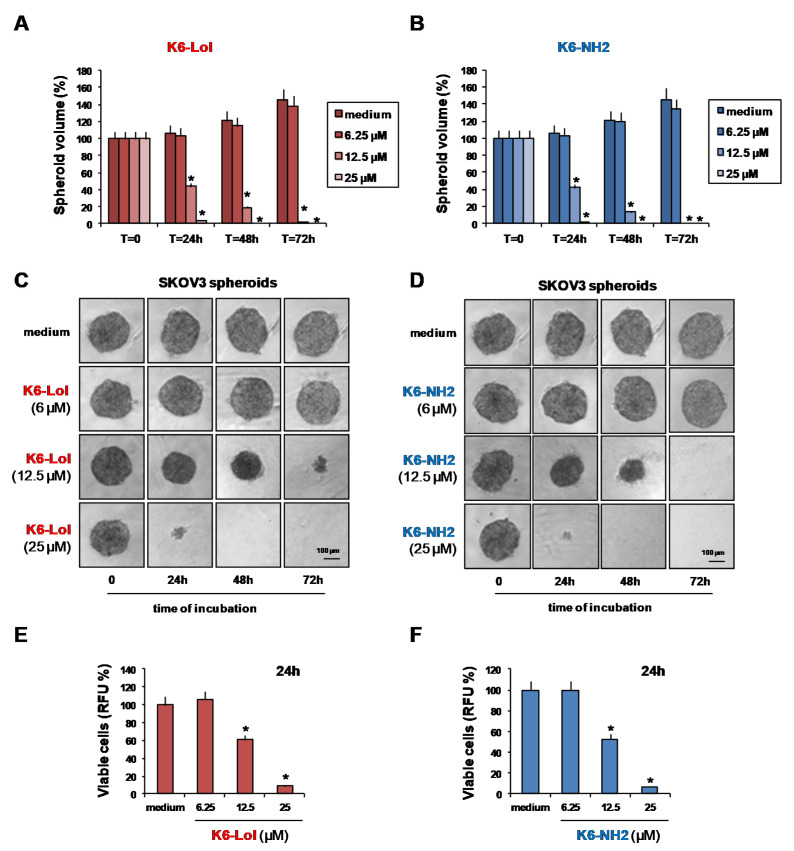
K6-Lol and K6-NH2 activity in SKOV3 spheroids (3D model). Four-day old SKOV3 single pre-formed spheroids were cultured in the absence or in the presence of (**A**) K6-Lol and (**B**) K6-NH2. Responses were evaluated by spheroid volume measurements. Values in the bar graphs are the mean and SD of the volume of at least five spheroids and three independent experiments. * *p* < 0.05 vs. medium. (**C**,**D**) Representative phase contrast micro-photographs showing SKOV3 spheroid volume decrease by (**C**) K6-Lol and (**D**) K6-NH2 treatment (original magnification 4×). (**E**,**F**) SKOV3 spheroid cell viability after 24 h treatment evaluated using PrestoBlue Cell Viability Reagent. Values are means and SD of three experiments each run in triplicate. * *p* < 0.05 vs. medium.

In OvCa, drug resistance can be intrinsic, acquired, or achieved by the aggregation of tumor cells as spheroids [13]. Peritoneal carcinomatosis with the formation of malignant ascites often characterizes late stage of OvCa. In ascitic fluid, OvCa cells exfoliate from the primary tumor, and aggregate to form OvCa stem cell-enriched MCTSs and heterospheroids [14,33], which contribute to drug resistance and spreading to secondary sites [33,34]. MCTSs, obtained by cultivation of tumor cells under non-adherent conditions, mimic tumor growth in ascitic fluid, resemble avascular micrometastases, and are thus considered effective three-dimensional (3D) first-line approaches for the screening of novel anticancer drugs [35,36]. Thus, to test peptaibols activity, we used SKOV3 cells, which are able to form large and dense spheroids and evaluated SKOV3-MCTS volume and cell viability (Figure 3). K6-Lol (Figure 3A) and K6-NH2 (Figure 3B) decreased the volume of SKOV3-MCTSs and completely eliminated them after 3 days of treatment at 25 µM (Figure 3A,B). Representative phase contrast micro-photographs, demonstrating the effects of K6-Lol (Figure 3C) and K6-NH2 (Figure 3D) on SKOV3-MCTS size, are shown. To further confirm these results, cell viability of SKOV3-MCTSs was evaluated after 24 h treatment with peptaibols using presto-blue cell reagent (Figure 3E,F). A dose-dependent decrease in SKOV3-MCTS cell viability with no significant differences in the cytotoxic effects of K6-Lol (Figure 3E) and K6-NH2 (Figure 3F) was detected.

### 2.5. Peptaibol Uptake by HRS and OvCa Cells

**Figure 4:**
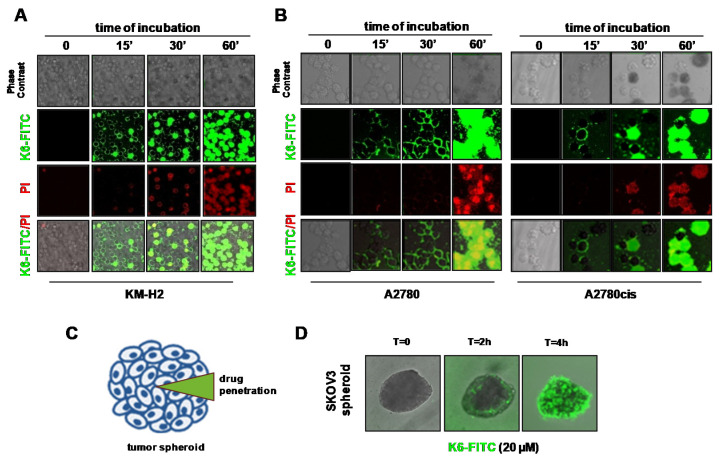
K6-FITC uptake by HL OvCa cells and OvCa spheroids. Representative confocal images showing (**A**) KM-H2 (**B**) A2780 (left panels) and A2780cis cells (right panels) double stained with K6-FITC (30 µM) (green) and propidium iodide (PI) (red) at various time points. (**C**) Illustration of drug penetration kinetic into spheroid. (**D**) Representative photomicrographs of a SKOV3 spheroid treated with K6-FITC (20 µM) and analyzed for K6-FITC uptake by confocal microscopy time-lapse.

Both peptaibols were rapidly taken up by cancer cells (Figure 4). The time-dependent uptake of K6 by tumor cells was demonstrated using K6-FITC and evaluated by confocal microscopy. Since K6-FITC was less active (IC_50_ ~ 30 µM) than K6-Lol or K6-NH2 (Supplemental Figure S6), we used K6-FITC at a concentration of 30 µM. To detect the uptake and the cytotoxic effects of K6-FITC, cells were double stained with peptaibols and propidium iodide (PI), a DNA intercalator not permeant to live cells with intact cell membranes. Since HDLM-2, HDLM-2dx, L-1236, and L-428 cells grow in suspension, for live cell imaging studies we used KM-H2 cells, which are capable of adhering to cell culture plates when seeded at low density. K6-FITC rapidly accumulated at the plasma cell membrane level before being internalized by KM-H2 cells (Figure 4A) and, after 60 min of incubation, all cells were double stained with PI (red fluorescence) and K6-FITC (Figure 4A), demonstrating the uptake and the rapid induction of cell death by K6-FITC. Similar experiments were performed with A2780 (Figure 4B, left panel) and its cisplatin-resistant clone A2780cis (Figure 4B, right panel). K6-FITC was rapidly taken up in both cell lines, then internalized, leading to cytotoxic effects (PI+ stained cells) (Figure 4B). Consistent with the dramatic cytotoxic effects of peptaibols, K6-FITC rapidly and deeply penetrated into SKOV3-MCTSs (Figure 4C,D).

Cytotoxic effects on MCTSs are usually obtained with drugs exhibiting high penetration and accumulation [37]. Our results suggest that both peptaibols, being capable of deeply penetrating and accumulating in MCTSs, could overcome the protective effects due to OvCa cell aggregation in ascitic fluid and easily penetrate in solid avascular micrometastases.

### 2.6. Peptaibols Activity in Healthy Cells

**Figure 5:**
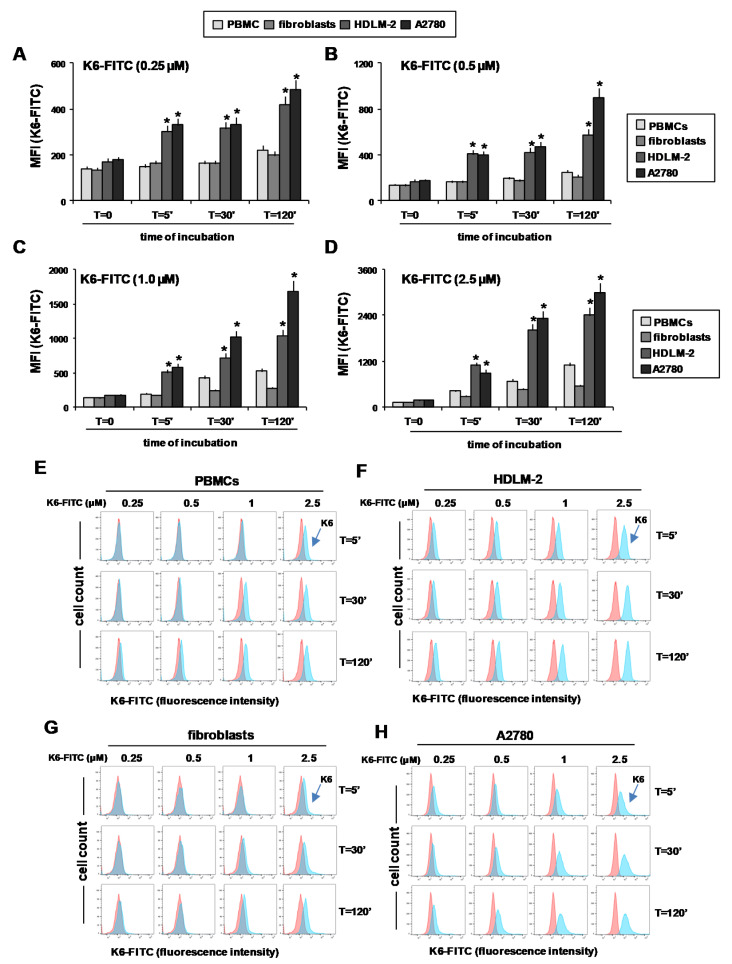
Comparison of K6-FITC uptake between normal healthy cells and tumor cells. (**A**–**D**) Mean fluorescence intensity (MFI) of normal healthy cells, peripheral blood mononuclear cells (PBMCs) and fibroblasts, tumor cells HDLM-2 and A2780 treated with (**A**) 0.25 µM, (**B**) 0.5 µM, (**C**) 1 µM, (**D**) 2.5 µM K6-FITC and analyzed by flow cytometry after 0, 5, 30 and 120 min of incubation. Bar charts are mean and SD of three independent experiments. * *p* < 0.05 vs. healthy cells (PBMNc and fibroblasts). (**E**–**H**) Representative cytofluorimetric histograms of (**E**) PBMCs, (**F**) HDLM-2, (**G**) fibroblasts, and (**H**) A2780 treated with different concentrations (0.25–2.5 µM) of K6-FITC for 5, 30, and 120 min.

A major obstacle to AMPs becoming anticancer drugs is their unwanted interactions with healthy cells. Therefore, we evaluated the cytotoxic effects of peptaibols (K6-Lol and K6-NH2) in human healthy peripheral blood mononuclear cells (PBMCs), and fibroblasts and compared their uptake of K6-FITC to that of tumor cells by flow cytometry (Figure 5). Peptaibols showed similar citotoxic effects in healthy cells, with IC_50_ of about 7 µM in PBMCs and 15 µM in fibroblasts, with respect to cancer cells (Table 1, Supplemental Figure S7). However, even if peptaibols exerted comparable cytotoxic activity, they could be less selective for normal cells than tumor cells due to the higher levels of the negative charged phosphatidylserine (PS) in tumor cell membranes with respect to healthy cells [38]. The higher PS content in cancer cells, by favoring the binding of positively charged peptaibols, could increase their selectivity towards tumor cells [39]. Consistently, we found that tumor cells (A2780 and HDLM-2) took up peptaibols in a higher quantity and in a faster way (mean fluorescence intensity of K6-FITC) than normal cells (PBMCs and fibroblasts) (Figure 5A–D). Representative flow cytometry histograms of K6-FITC uptake by PBMCs (Figure 5E), HDLM-2 (Figure 5F), fibroblasts (Figure 5G), and A2780 (Figure 5H) at different times and concentrations are shown in Figure 5E–H.

### 2.7. Peptaibols Cause Cell Membrane Permeabilization in Tumor Cells

**Figure 6:**
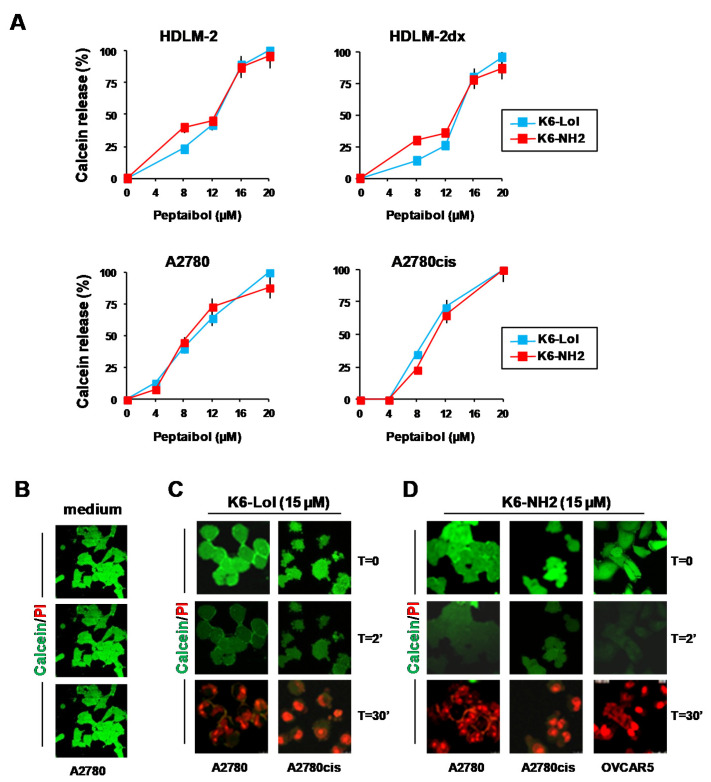
K6-Lol and K6-NH2 rapidly induce calcein release and cell death in OvCa cells. Membrane permeability assays. (**A**) To monitor the lack of the cell membrane integrity after peptaibols treatment, tumor cells were prestained with calcein AM. Then, cells were treated with peptaibols (0–20 µM) and fluorescent calcein in supernatants was evaluated immediately (2 min). Results are expressed as the percentage of calcein released by cancer cells with respect to cells disrupted with triton X (100%, total fluorescent calcein converted to a green fluorescent form by cancer cells). Bar charts are mean and SD of three independent experiments. (**B**–**D**) Cell-based calcein release assay evaluated by confocal microscopy. OvCa cells were preincubated with calcein AM (green staining), then stained with PI (to evaluate dead cells) (red staining) and treated with peptaibols. (**B**) Then, untreated cells (medium) and (**C**,**D**) cells treated with peptaibols (15 µM) were monitored for calcein AM release by real-time fluorescence using the Operetta system.

The antimicrobial mechanism of peptaibols has been extensively studied, and it has been demonstrated that their abilities to induce cell death are essentially related to cell membrane permeabilization mediated by their pore-forming capabilities [40]. Trichogin GA IV links and then deeply penetrates into the membrane core [41], modifying membrane integrity and rapidly inducing cell membrane lysis without interacting with mitochondria [6]. Thus, we investigated whether our peptaibols were able to induce cell membrane permeabilization in cHL and OvCa cells, using the calcein AM assay [42]. Calcein AM is a non-fluorescent, hydrophobic compound that easily penetrates intact and living cells. After internalization, calcein AM is hydrolyzed by intracellular esterases, producing a hydrophilic, strongly fluorescent compound that is retained in the cell cytoplasm and released in the presence of cell membrane pores. We evaluated the fluorescent calcein AM released in culture medium from cHL and OvCa cells treated or not with peptaibols. Incubation of calcein-treated cHL cells (HDLM-2 and HDLM-2dx) and OvCa cells (A2780 and A2780cis) with peptaibols determined an immediate and dose-dependent release of calcein AM (green fluorescence) (Figure 6A), that was complete at 20 µM concentration of peptaibols (Figure 6A). Both K6-Lol and K6-NH2 determined comparable calcein AM release in drug-sensitive (A2780 and HDLM-2) and drug-resistant cancer cells (A2780cis and HDLM-2dx) (Figure 6A). The capability of peptaibols to immediately release calcein AM, and then to induce cytotoxic effects, was further demonstrated by time-lapse confocal microscopy (Figure 6B–D).

We used OvCa cells labeled with calcein AM (green fluorescence) and Propidium Iodide (PI) (red fluorescence) to evaluate cell membrane permeabilization and the cytotoxic effects induced by peptaibols (dead cells), respectively. Untreated cells (medium only) (left panel) did not release calcein AM and PI staining was absent (Figure 6B). Conversely, OvCa cells treated with K6-Lol (Figure 6C) and K6-NH2 (Figure 6D) immediately released the green fluorescent calcein AM and, after 30′, we observed cell death (PI stained cells) only in peptaibol-treated cells (Figure 6C,D).

Taken together, these results demonstrate that both peptaibols very quickly lead to membrane lysis and, consequently, to cell death.

### 2.8. Peptaibols Induce Phosphatidylserine Exposure wihout Caspase Activation

**Figure 7:**
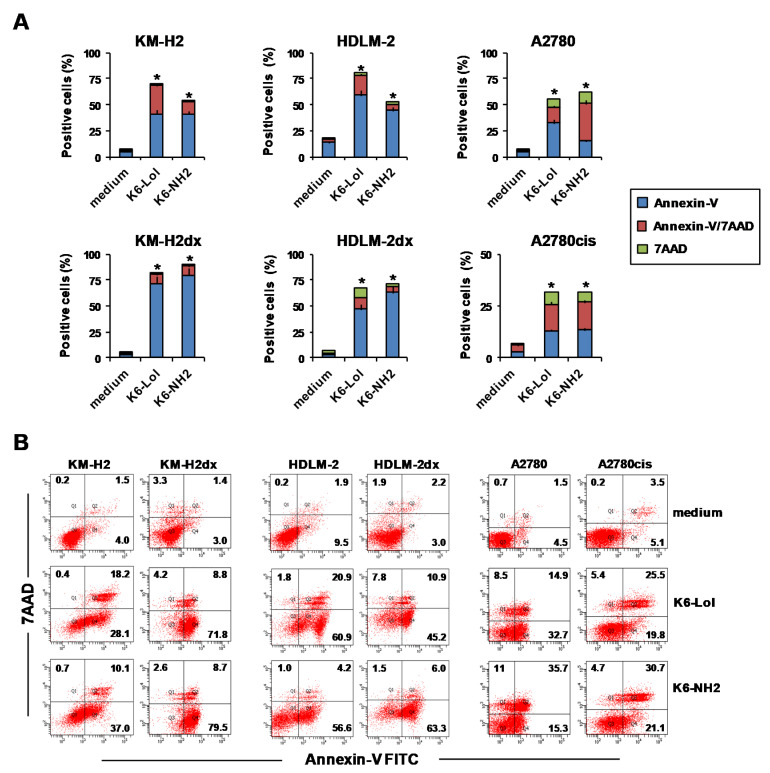
Annexin-V staining after peptaibol treatment. HRS cells (KM-H2, KM-H2dx, HDLM-2, and HDLM-2dx) and OvCa cells (A2780 and A2780cis) were treated with K6-Lol and K6-NH2 for 24 h. Then cells were double stained with Annexin-V-FITC and 7AAD and analyzed by flow cytometry. (**A**) Bar charts are the mean of three independent experiments showing the percentage of annexin-V and 7AAD positive cells. * *p* < 0.05 vs. medium. (**B**) Representative cytofluorimetric dot plots of Annexin-V-FITC and 7AAD staining.

The exposure of phosphatidylserine (PS) on the outer plasma membrane is considered a marker of apoptosis and Annexin-V has a very high affinity for the negatively charged PS. In our experimental models, after 24 h treatment with an IC_50_ dose of peptaibols, we found Annexin-V+ stained cells (Figure 7A,B) without caspase (3, 8 and 9) activation (no green fluorescent signal), neither in HRS cells (Supplemental Figure S8) nor in OvCa cells (Supplemental Figure S9), excluding apoptosis as a mechanism of action of peptaibols. Representative flow cytometry dot plots of tumor cells treated with peptaibols and stained with Annexin-V-FITC and 7AAD, as a marker of cell death, are shown in Figure 7B. Representative flow cytometry histograms showing caspase activation by peptaibols in HRS cells, OvCa cells, and by cisplatin in HDLM-2 cells (positive control of caspase activation) are shown in Supplemental Figures S8 and S9.

A possible explanation for these conflicting results, i.e., PS exposure (marker of apoptosis) in the absence of caspase activation, is that the early cell membrane permeabilization by peptaibols could allow the diffusion of the Annexin-V, which can then bind PS on the inner leaflet. Alternatively, membrane insertion of peptaibols could change the lipid position in the asymmetric bilayer, allowing the translocation of PS to the outer leaflet and its exposure on membrane surface, where it then binds the Annexin-V. In both scenarios, peptaibols treated cells stain positive for Annexin-V, but finally exert fast cytotoxic activity by membrane lysis.

**Figure 8:**
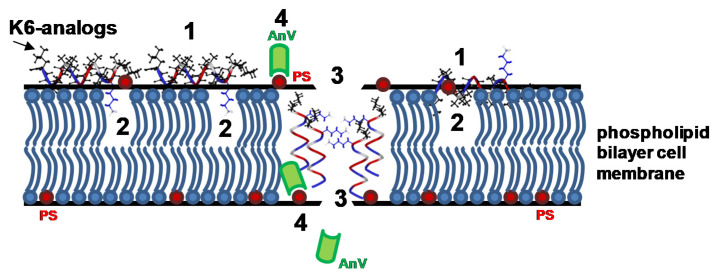
Proposed mechanism of peptide–membrane interaction of K6-analogs. (**1**) The Lys residue at position 6 of K6-analogs (in black) could interact with the hydrophilic heads of phospholipid membranes (**2**) promoting helix insertion (**3**), leading to cell membrane permeabilization by pore formation and (**4**) Annexin-V staining. Phosphatidylserine (PS) (in red) could translocate and appear in the external membrane and bind to Annexin-V (AnV) (in green), or Annexin-V could diffuse and then bind PS on the inner leaflet.

A proposed mechanism of peptide–membrane interaction of K6-analogs with cell membranes is shown in Figure 8.

### 2.9. Peptaibols Maintain a Stable Helical Structure in the Presence of Cancer Cells

**Figure 9:**
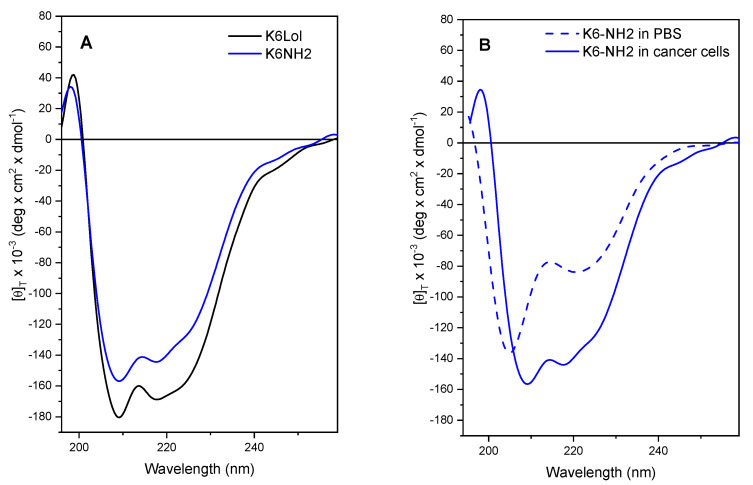
Circular dichroism profiles of K6-Lol and K6-NH2 in the presence of cancer cells. (**A**) L-428 cells were treated with peptaibols and the peptide 3D structure was analyzed by CD. The two peptaibols adopt a stable helical conformation (peptide concentration: 10 µM). (**B**) Comparison between the CD profiles acquired for K6-NH2 in the presence (full line) or absence (dashed line) of cancer cells. The increase in the intensity of the band at about 220 nm for the peptide in the presence of cancer cells (panel B, full line) is indicative of the onset of peptide aggregation in cancer cells and not in PBS buffer alone.

To monitor changes in the structure of peptaibols during their interactions with cancer cells, we performed a conformational analysis of both K6-Lol and K6-NH2 by circular dichroism (CD) in the presence of cHL-derived cell line L-428 (peptaibol concentration, 10 µM). To avoid light scattering, the cells (2×10^5^ cells/mL) were suspended in a PBS buffer. The array of chiral molecules in cells created a significant background signal, but that did not hamper the detection of the peptide signal in the presence of cancer cells, which was clearly visible (see Supplemental Figure S10). The CD profiles reported in Figure 9 are diagnostic of the presence of a well-developed, right-handed, α-helical conformation. Peptaibols are known for their stable helices, due to the presence of the helix-inducer α-amino acid residue Aib in their sequence. To the best of our knowledge, this is the first time that the stability of their 3D structure is proved in the presence of human cells. The presence of a stable helix is at the basis of peptaibol interaction with membranes. Figure 9B reports the CD spectra acquired for K6-NH2 in PBS buffer with or without cancer cells. The increase in intensity of the absorption band centered at about 222 nm, registered for K6-NH2 in the presence of cancer cells (Figure 9B), is diagnostic of aggregation between peptide helices. Such helical bundles are needed for efficient peptaibol–membrane interaction. Their formation is the first step towards pore formation. The stability of peptaibol helical structures in the presence of cancer cells—proven by CD even after a few days (data not shown)—likely plays a key role in their anticancer activity as well, especially against the 3D SKOV3 spheroids. The routine application of CD analysis in the presence of cancer cells, as herein described, would make it easier to build solid structure–activity relationships. Besides, as stated above, it also gives indirect information on peptide stability in the biological environment.

## 3. Materials and Methods

### 3.1. Materials

Fmoc-protected amino acids and solvents for peptide synthesis were purchased from Sigma-Aldrich (Milano, Italy). Oxyma-pure and diisopropylcarbodiimide (DIC) were acquired from IRIS Biotech. Doxorubicin (doxo) (Pfizer Italia, Milano, Italy) and cisplatin (CDDP), (Mayne Pharma, Naples, Italy) were surplus drugs from the clinical pharmacy of CRO Aviano.

### 3.2. Peptide Synthesis

We synthesized the peptaibol analogs K6-Lol and K6-NH2, and the fluorescent K6-FITC, through the semiautomatic peptide synthesizer Biotage MultiSynTech. The protocol for the solid-phase peptide synthesis (SPPS) of the peptides involves the use of the ecofriendly active agents Oxyma pure (ethyl cyano(hydroxyimino) acetate) and DIC [43]. All steps were carried out using ethyl acetate/dimethylsulfoxide 9:1 mixture as solvent, to avoid the use of N,N-dimethylformamide (DMF). We used only 2 equivalents of the incoming, activated amino acid residue for each coupling, except for the step involving an Aib residue as the nucleophile. In this latter case, the incoming residue was successfully inserted by means of a double coupling, using 2 and 1 equiv., respectively. The analog terminating with the naturally occurring leucinol was synthesized on a 2-chlorotrytil resin preloaded with the 1,2-amino alcohol, while K6-NH2 was obtained on a Rink amide resin. We grew the C-terminal free amine precursor of K6-FITC on a preloaded 2-chlorotrytil resin. The fluorescein isothiocyanate was linked at the C-terminus in solution. After cleavage from the resin, the crude peptides were already >90% pure. They were purified to >99% by means of medium-pressure chromatography on a Biotage Isolera Prime instrument. Final yield was 85% and 91% (for K6-Lol and K6-NH2, respectively). Peptide identification and purity check were performed by high resolution mass spectrometry, HPLC and 1D NMR. The full characterization of the peptides is reported in the supporting information (Supplemental Figures S11–S17).

### 3.3. Serum Stability Assays

Peptides were dissolved in dimethylsulfoxide to a concentration of 5 g/L. Quantities of 40 μL of each peptide solution in DMSO were added to a vial containing 1 mL of Hepes buffer (pH 7.2) and 250 μL of previously thawed human serum kept at 37 °C in a thermal bath. Immediately after this step, 100 μL were taken from this mixture and transferred to an eppendorf containing 200 μL of ethanol, and placed on ice for at least 15 min. This sample represents the zero time of the experiment. The mixture was then sampled at 5 min, 15 min, 30 min, 1 h, 1.5 h, 2 h, 3 h and 24 h from the beginning of the experiment. The samples were centrifuged for 5 min at 13,000 rpm to allow the serum components to precipitate, the supernatant was recovered, and the degradation trend was analyzed by HPLC. To check the retention times of the peptides analyzed and to verify that the peptides were stable in the buffer itself, a reference was prepared that did not contain human serum. The treatment of this reference sample was the same as that used for the samples containing serum described above, but only three samples were taken: at time zero, after 3 h and after 24 h. Finally, to verify the effectiveness of the human serum used, the degradation of a model peptide, XP-1, of which the degradation times are known, was followed every day.

### 3.4. Proteolytic Stability Assays

Peptide stability in the presence of either trypsin or pronase (proteases from *Streptomyces griseus*, purchased from Sigma Aldrich) was tested. Briefly, peptides were dissolved in the buffer solution prepared according to standard procedure (tris(hydroxymethyl)aminomethane hydrochloride (TRIS·HCl) 20mM, CaCl_2_ 20mM (pH 7.6) for pronase; TRIS·HCl 10mM (pH 7.8) for trypsin). Each enzyme was added to the peptide in a *w/w* ratio of 1:100 and the mixture incubated for 4 h at 37 °C. HPLC analysis on a C18 column (Phenomenex Jupiter 5 μm, 300 Å, 250 × 4.5 mm) was performed at intervals. The gradient used was 70–100% B in 10 min; eluent A H_2_O/CH_3_CN 9:1 with 0.05% trifluoroacetic acid (TFA); eluent B CH_3_CN/H_2_O 9:1 with 0.05% TFA. A reference peptide, known to be sensitive to proteolysis by those enzymes, was used as positive control, to check enzyme performance.

### 3.5. Circular Dichroism (CD)

The CD spectra were obtained on a Jasco (Tokyo, Japan) J-715 spectropolarimeter. Fused quartz cells (Hellma) of 1-mm path length were used. The values are expressed in terms of [θ]_T_, total molar ellipticity (deg∙cm^2^∙dmol^−1^). Spectrograde methanol and trifluoroethanol (TFE) (Acros, Geel, Belgium) were used as solvents. The CD measurements in membranes were carried out with a fused quartz cell of 0.5-mm pathlength (Hellma, Mühlheim, Germany). To suppress scattering while acquiring CD spectra of peptides in the presence of cancer cells, phosphate buffer was used as medium.

### 3.6. Cell Culture

Authenticated cHL-derived cell lines L-1236, L-428, KM-H2, and HDLM-2 were from DSMZ (Braunschweig, Germany). Doxorubicin-resistant KM-H2dx and HDLM-2dx cells were developed in our laboratory by continuous exposure of KM-H2 and HDLM-2 cells to 35 nM doxorubicin. HDLM-2dx and KM-H2dx cells were maintained in doxorubicin and cultured without the drug 72 h before use. All cell lines are here collectively called “cHL cells”. Authenticated OvCa cell lines A2780 and A2780cis were from Sigma-Aldrich, (Milano, Italy) SKOV3 (HTB-77) were obtained from the American Type Culture Collection (ATCC), OVCAR5 (NIH) were provided by Dr. Baldassarre (CRO, Aviano, Italy). A2780cis cells were maintained in 1 µM cisplatin and cultured without the drug for 72 h before use. All cell lines were routinely tested for mycoplasma, with negative results, and authenticated in our laboratory using PowerPlex 16 HS System (Promega) and GeneMapper ID version 3.2.1 to identify DNA short tandem repeats. cHL and OvCa cell lines were cultured in RPMI-1640 medium (Gibco, Thermo Fisher Scientific, Monza, Italy) containing 10% fetal bovine serum (Gibco), called “complete medium”. Peripheral blood mononuclear cells (PBMCs) were obtained from healthy donor blood by density gradient centrifugation on a Ficoll-Paque PLUS (GE Healthcare); they were washed twice with PBS before use and cultured in RPMI-1640 medium containing 10% fetal bovine serum. Human dermal fibroblasts, a generous gift of Dr. Capuano [44], were maintained in Dulbecco′s Modified Eagle′s Medium (DMEM) high glucose (Gibco) plus 10% fetal bovine serum.

### 3.7. Cellular Assays

To determine the cytotoxicity of K6-Lol and K6-NH2, cHL cells and PBMCs (2.0 × 10^5^/mL) were seeded in 96-well plates in complete medium and treated with increasing concentrations of peptaibols (0–40 µM) in triplicate. After 24 h treatment, cell viability was evaluated using the CellTiter 96^®^ AQueous One Solution Cell Proliferation Assay (MTS) (Promega, Milano, Italy) and revealed using a computer-interfaced GeniusPlus microplate reader (Tecan). OvCa cells and fibroblasts were seeded in 96 well (10.0 × 10^3^) the day before treatment to allow cell adherence. After 24h, cells were treated with increasing concentrations of peptaibols (0–40 µM) in triplicate. Cell viability was assessed by 3-(4,5-dimethylthiazol-2-yl)-2,5-diphenyltetrazolium bromide (MTT) (Sigma Aldrich) assay and revealed with Tecan. Half maximal inhibitory concentrations (IC_50_) were calculated using CalcuSyn software, v2.1 (Biosoft, Ferguson, MO, USA) [45]. Fold resistance (FR) was calculated as the ratio of the IC_50_ of drug-resistant cell line (KM-H2dx, HDLM-2dx, and A2780cis) to that of the respective parental cell line (KM-H2, HDLM-2, and A2780).

To obtain the 3 dimensional (3D) SKOV3-MCTSs, cells were cultured in non-adherent conditions on plates coated twice with 20 mg/mL of poly(2-hydroxyethyl methacrylate) (poly-HEMA; Sigma) in 95% ethanol. Single spheroids with defined size were generated by culturing 1.0 × 103 SKOV3 cells onto poly-HEMA-coated 96-well for 4 days [46]. Then, single SKOV3-MCTSs were treated with increasing concentrations of peptaibols (0–25 µM). SKOV3-MCTSs size was measured and photographed up to 72 h after treatment initiation using an inverted microscope (Eclipse TS/100, Nikon) with the DS Camera Control Unit DS-L2 photo micrographic system. SKOV3-MCTSs volumes were calculated using the formula: (width^2^ × length × 3.14)/6 [47].

Multiple SKOV3-MCTSs were obtained by seeding 10.0 × 10^3^ cells in non-adherent conditions. After 24 h incubation with peptaibols, cell viability was evaluated using PrestoBlue Cell Viability Reagent (Thermo Fisher Scientific) and revealed by Tecan.

### 3.8. K6- FITC Uptake and Annexin-V Assay

The uptake of peptaibols was evaluated by confocal microscopy and flow cytometry using the fluorescent K6-FITC analog [6]. For confocal microscopy, cells were plated onto glass bottom culture dishes and treated with K6-FITC (30 µM) and propidium iodide (PI) 2 µg/mL. K6-FITC uptake was monitored using confocal microscope in time-lapse xyzt acquisition mode (Leica DM IRE2) keeping cells at 37 °C and 5% CO_2_. To trace peptaibol penetration in spheroids, single four-day-old SKOV3-MCTs were incubated with K6-FITC (20 µM) and images acquired using confocal microscopy.

For flow cytometry, cells were treated with different concentrations of K6-FITC (0–2.5 µM). After 5, 30 and 120 min of incubation, cells were washed and evaluated for green fluorescence intensity by flow cytometry on a BD FACSCanto II flow cytometer. Annexin-V assay: cells were treated with peptaibols for 24 h, then stained for 15 min with FITC AnnexinV (Thermo Fisher Scientific, Monza, Italy) and 7-Amino-Actinomycin D (7AAD) (BD Pharmingen, Milano, Italy) and analyzed using flow cytometry. Data were analyzed using BD FACSDiva v.8.0.1 software (BD Biosciences, Milano, Italy).

### 3.9. Calcein AM Assay

cHL cells (2.0 × 10^5^/mL) were stained with cell-permeant calcein AM dye (Invitrogen) (2 µM) for 30 min, washed and seeded in 96-well plates. OvCa cells (10.0 × 10^3^) were seeded in 96-well plates the day before the assay to allow cell adherence, stained for 30 min with calcein AM and then washed. To evaluate the effect on cell membrane permeabilization due to peptaibol treatment, calcein-stained cells were treated with different concentrations of K6-Lol or K6-NH2. Calcein AM release in culture medium was monitored immediately (2 min) using a Tecan spectrofluorometer microplate reader. Complete, maximum release of calcein (control, 100%) was obtained by treating the cells with 1% Triton X-100 (Fluka, Life Technologies, Monza, Italy). Calcein AM release was monitored also by confocal microscopy. Briefly, OvCa cells stained with calcein AM (2 µM) were washed, then stained with PI (2 µg/mL) and treated with K6-Lol or K6-NH2 (15 µM). Calcein AM release (i.e., decrease in green fluorescence in cancer cells) was monitored by confocal microscopy in time lapse xyzt acquisition mode.

### 3.10. Statistical Analysis

Statistical analysis was carried out using GraphPad Prism version 6.0 software (GraphPad, La Jolla, CA, USA). Student’s t-test was used to compare two groups. One-way ANOVA followed by Dunnett’s test was used to compare each of a number of treatments with a single control. A *p*-value < 0.05 was considered significant.

## 4. Conclusions

We synthesized analogs of the natural peptaibol trichogin GA IV that had a Lys-for-Gly substitution at position 6 and three different C-terminal moieties with good yield and purities, following a protocol that was in line with the guidelines to achieve green peptide synthesis. The change from the naturally occurring 1,2-aminoalcohol to a C-terminal amide reduced the production cost considerably (we calculated a reduction of more than 50%, thanks to the change of the resin).

The analogs are fully resistant to degradation both in plasma and in the presence of proteolytic enzymes. Despite their short length (11 residues), the peptides were found to adopt a very stable helical conformation even in the presence of cancer cells.

These new peptaibols exert comparable cytotoxic effects against various cancer cell lines, including tumor cells with acquired resistance to doxorubicin and cisplatin, and OvCa cells cultured as 3D-MCTSs. Peptaibols are rapidly taken up by 2D models of cHL and OvCa cells, leading to cell membrane permeabilization and rapid cell death; moreover, they deeply penetrate and kill OvCa SKOV3-MCTSs.

Peptaibols show similar cytotoxic effects in both cancer and normal cells but are taken up more rapidly by cancer cells than normal healthy cells. Their interactions with the tumor cell membrane induce PS externalization but not apoptosis. Since the two peptaibols demonstrate equivalent biological activity, K6-Lol can be safely replaced by the less expensive K6-NH2.

## Figures and Tables

**Table 1:** Half-maximal inhibitory concentrations (IC_50_) with the related standard deviation (SD) of the peptaibols K6-Lol, K6-NH2, the reference drugs cisplatin and doxorubicin: in HL cell lines HDLM-2, KM-H2, their doxorubicin-resistant clones KM-H2dx and HDLM-2dx, L-1236, L-428 cells; in OvCa cell line A2780 and its cisplatin-resistant clone A2780cis, OVCAR5 and SKOV3 cells; in human peripheral blood mononuclear cells (PBMCs) and human fibroblasts.

Cell Line	Compound (IC_50_) ^1^			
	K6-Lol (µM)	K6-NH2 (µM)	Cisplatin (µM)	Doxorubicin (nM)
HDLM-2	12.6 (1.1)	13.2 (1.2)	1.3 (0.1)	40.5 (3.7)
HDLM-2dx	11.9 (1.0)	12.6 (1.0)	4.0 (0.4)	368.1 (34.1)
KM-H2	7.5 (0.7)	7.8 (0.6)	0.5 (0.0)	22.1 (1.9)
KM-H2dx	7.2 (0.6)	8.2 (0.7)	1.2 (0.1)	202.4 (19.8)
L-1236	11.7 (1.0)	9.4 (1.1)	0.8 (0.1)	46.4 (4.1)
L-428	8.9 (0.9)	9.5 (1.0)	0.5 (0.1)	18.4 (1.4)
A2780	7.9 (0.8)	8.5 (0.9)	1.3 (0.1)	20.4 (1.8)
A2780cis	9.1 (0.8)	9.0 (0.9)	10.4 (0.9)	115.5 (10.4)
OVCAR5	7.5 (0.6)	7.5 (0.6)	7.7 (0.7)	470 (41.9)
SKOV3	8.4 (0.8)	10.5 (1.1)	7.7 (0.8)	130.4 (11.7)
PBMCs	7.1 (0.4)	7.9 (0.4)	nd	nd
Fibroblasts	15.0 (1.4)	15.5 (1.4)	nd	nd

^1^ Mean (SD are reported in brackets).

## Data Availability

The data presented in this study is contained within the article and supplementary material.

## Supplementary Materials

The following are available online at https://www.mdpi.com/article/10.3390/ijms22168362/s1.
