# Supplementary material for: Analogs of a Natural Peptaibol Exert Anticancer Activity in Both Cisplatin- and Doxorubicin-Resistant Cells and in Multicellular Tumor Spheroids
Source: Int J Mol Sci. 2021 Aug 4;22(16):8362. doi: 10.3390/ijms22168362 (PMC8394784; doi:10.3390/ijms22168362)
Supplement: Supplementary file 1 [file ijms-22-08362-s001.zip › ijms-1300402-supplementary.pdf]

SUPPORTING INFORMATION

Analogs of a natural peptaibol exert anticancer activity in both cisplatin- and doxorubicin-resistant cells and in multicellular tumor spheroids

Naike Casagrande[1], Cinzia Borghese[1], Laura Gabbatore[2], Laura Morbiato[2], Marta De Zotti[2,*], Donatella Aldinucci[1,*]

Affiliations

[1]Molecular Oncology, Centro di Riferimento Oncologico di Aviano (CRO) IRCCS, Italy

[2] Department of Chemistry, University of Padova, 35131, Padova, Italy.

*These Authors are colast and cocorresponding Authors

Correspondence *marta.dezotti@unipd.it and daldinucci@cro.it

Supplemental Figure S1

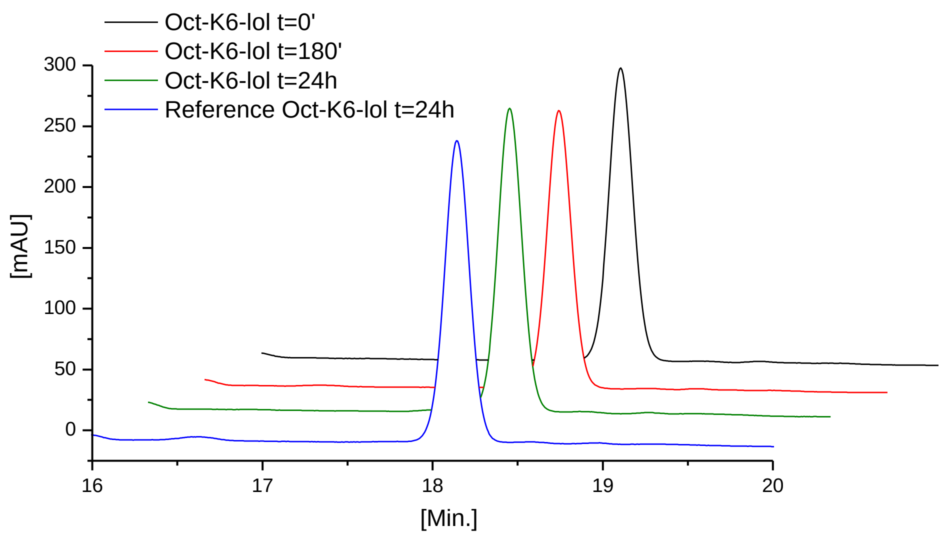

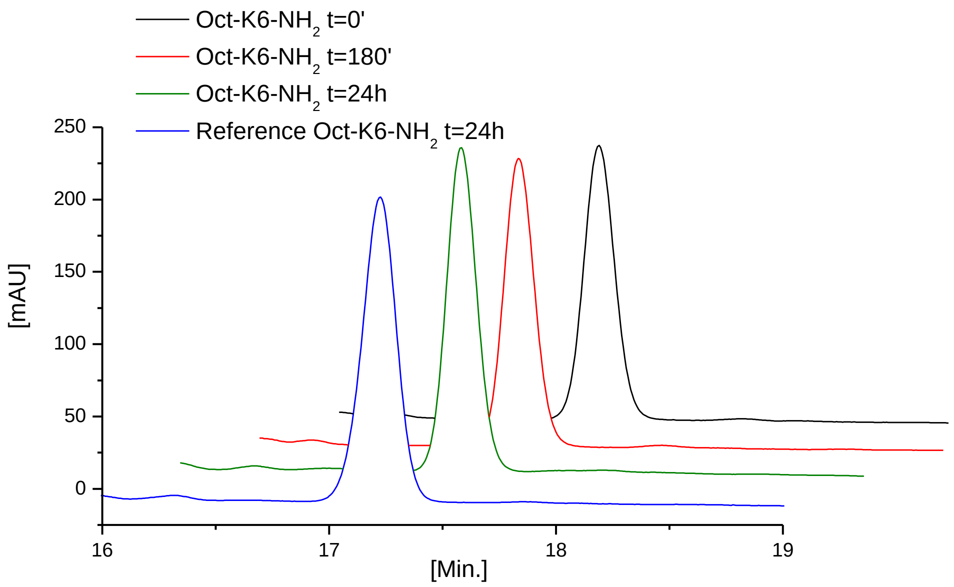

Supplemental Figure S1. K6-Lol and K6-NH2 in the presence of human serum. K6-Lol (**top panel**) and K6-NH2 (**bottom panel**) in the presence of human serum after 0, 3 and 24 hours incubation. Chromatograms at 215 nm. A slight offset was applied to the spectra on the x-axis for clarity. he HPLC chromatograms show that they are resistant for longer than 24 hours. To both confirm the retention times and verify that the peptides are stable in the buffer itself, the HPLC spectra of the peptides in serum-free buffer were also acquired after 24 hours.

Supplemental Figure S2

A

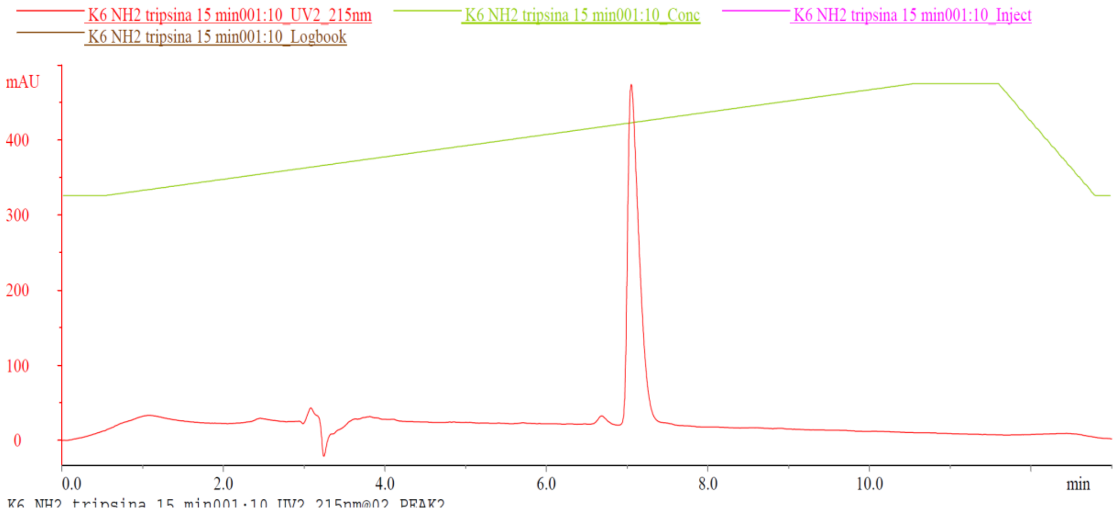

B

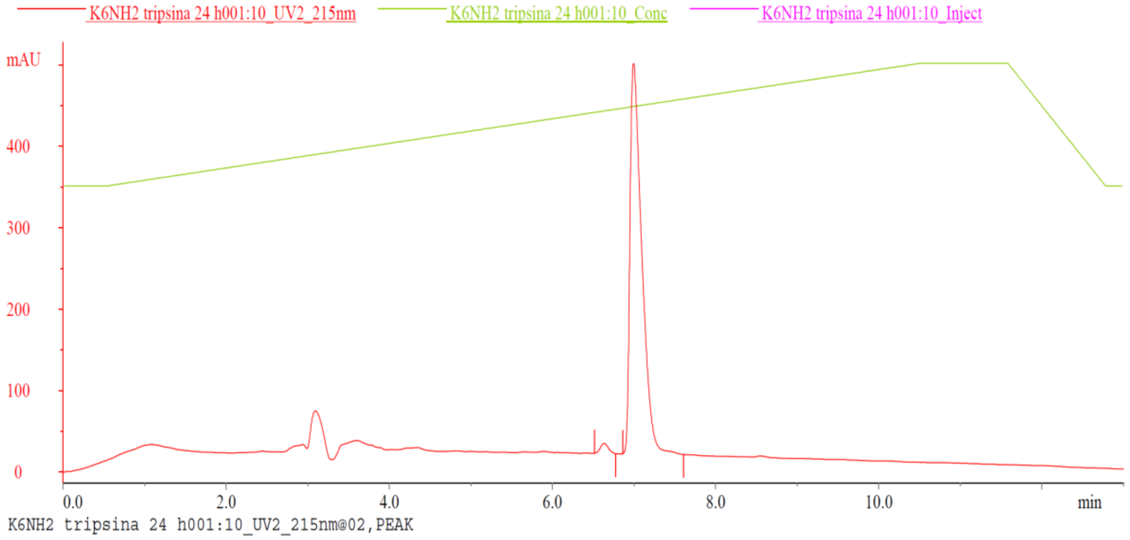

Supplemental Figure S2. Chromatograms of K6-NH2 in the presence of trypsin. K6-NH2 in the presence of trypsin after 15 minutes (**A**) and 24 hours (**B**) incubation. Chromatograms at 215 nm. The control has been digested within 15 minutes under the same experimental conditions.

Supplemental Figure S3

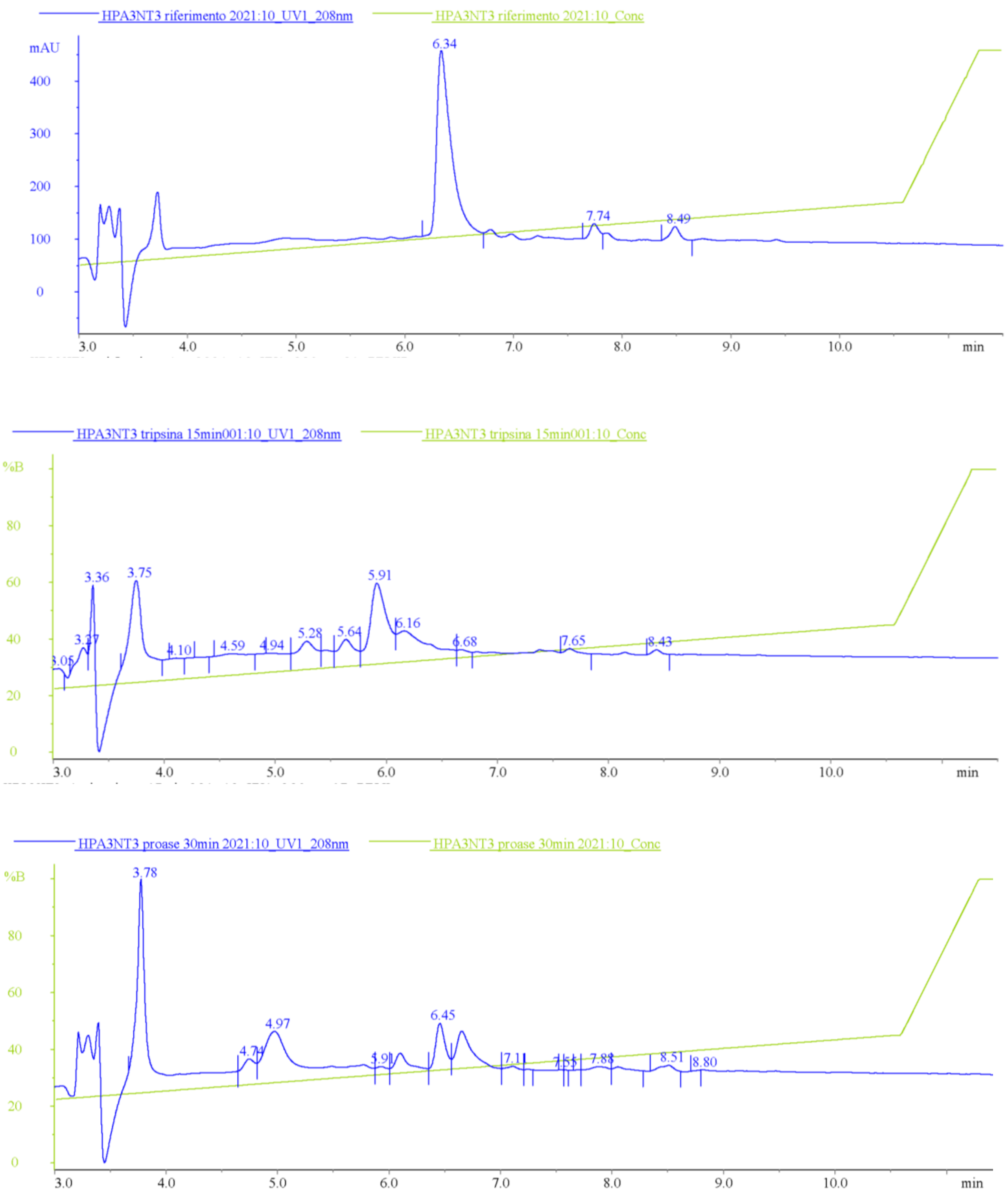

Supplemental Figure S3. Reference peptide in the presence of pronase and trypsin. The reference peptide (control) (**top panel**, peptide in buffer) has been totally digested within 15 minutes in the presence of trypsin (**middle panel**) and within 30 minutes in the presence of pronase (**bottom panel**).

Supplemental Figure S4

A

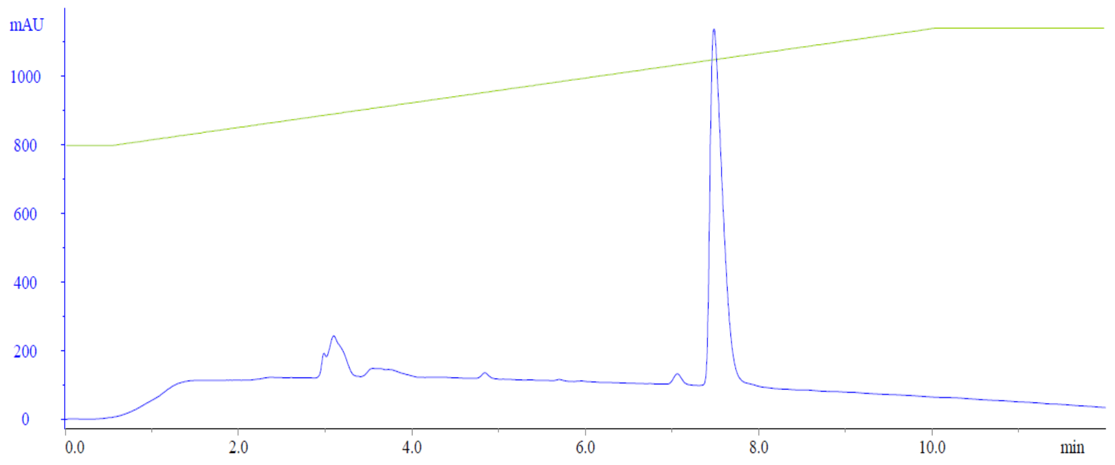

B

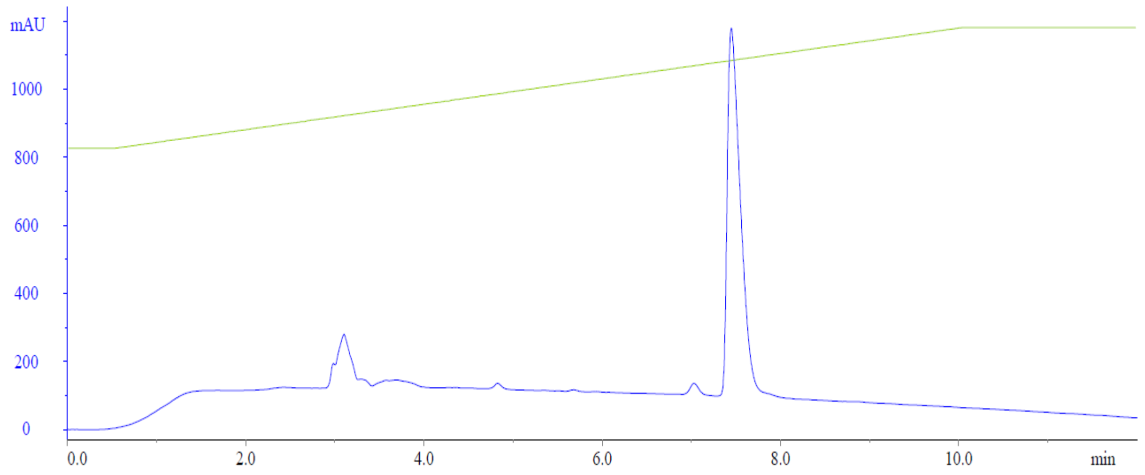

Supplemental Figure S4. Chromatograms of. K6-NH2 in the presence of pronase. K6-NH2 in the presence of pronase after 15 minutes (**A**) and 24 hours (**B**) incubation. Chromatograms at 210 nm. The control has been digested within 15 minutes under the same experimental conditions.

Supplemental Figure S5

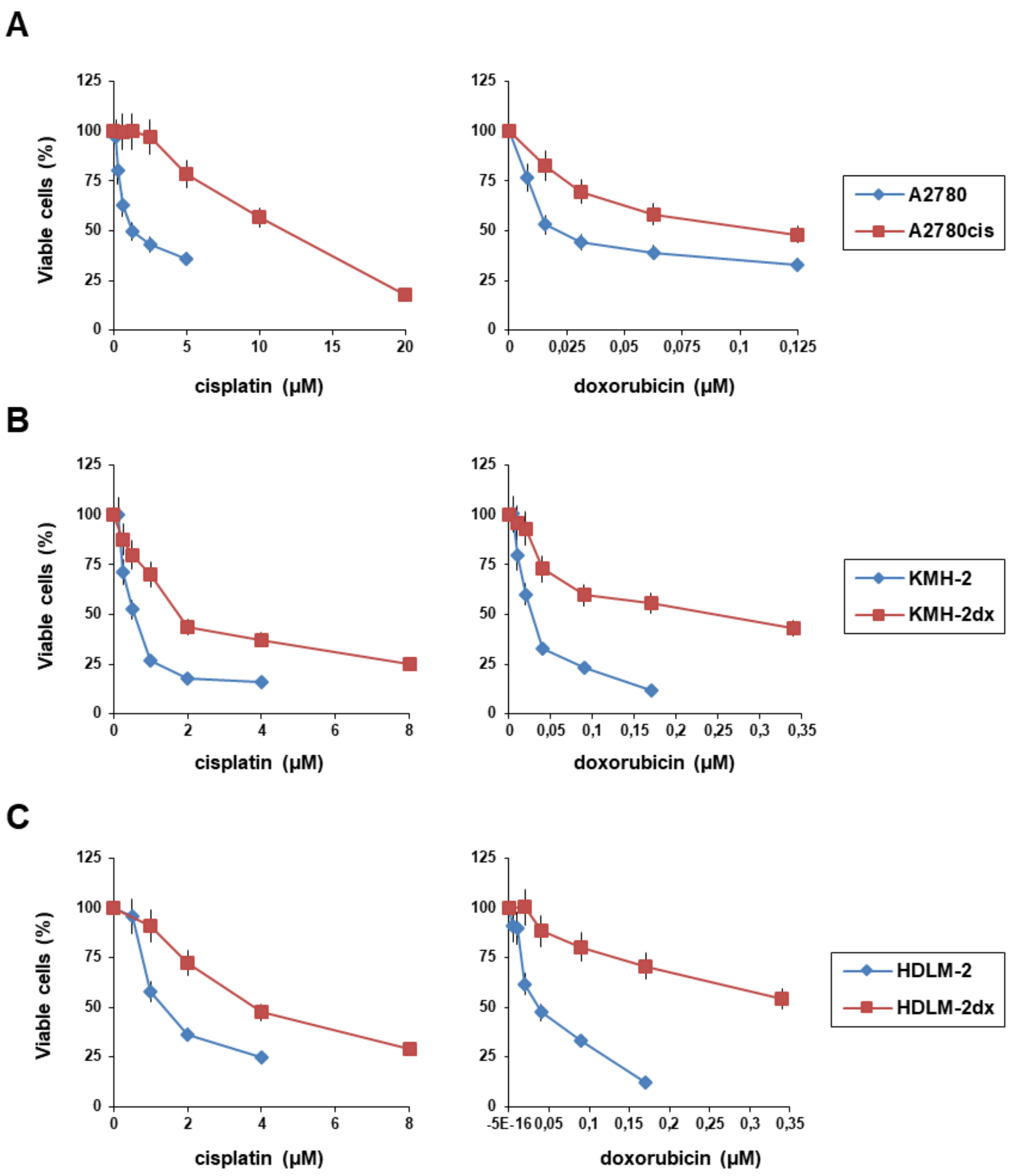

Supplemental Figure S5. Cytotoxic activity of cisplatin and doxorubicin in A2780, KM-H2, HDLM-2 and their drug-resistant clones. Tumor cells were exposed to increasing concentrations of drugs. (**A**) After 24 hours, cell viability was evaluated by MTT assay for adherent cells A2780 and A2780cis**,** (**B, C**) and by MTS assay for KM-H2, KM-H2dx, HDLM-2 and HDLM-2dx cells. Results are mean and SD of three independent experiments.

Supplemental Figure S6

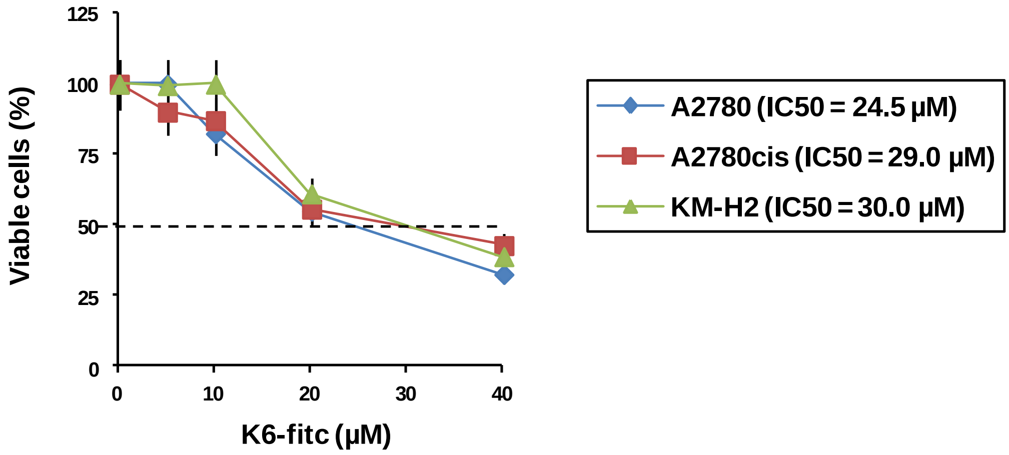

Supplemental Figure S6. Cytotoxic activity of K6-FITC. A2780, A2780cis, and KM-H2 cells were cultured with K6-FITC. After 24 hours cell viability was evaluated by MTT assay for A2780 and A2780cis and by MTS assay for KM-H2 cells. Results are mean and SD of three independent experiments. IC_{50} was calculated using Calcusyn software.

Supplemental Figure S7

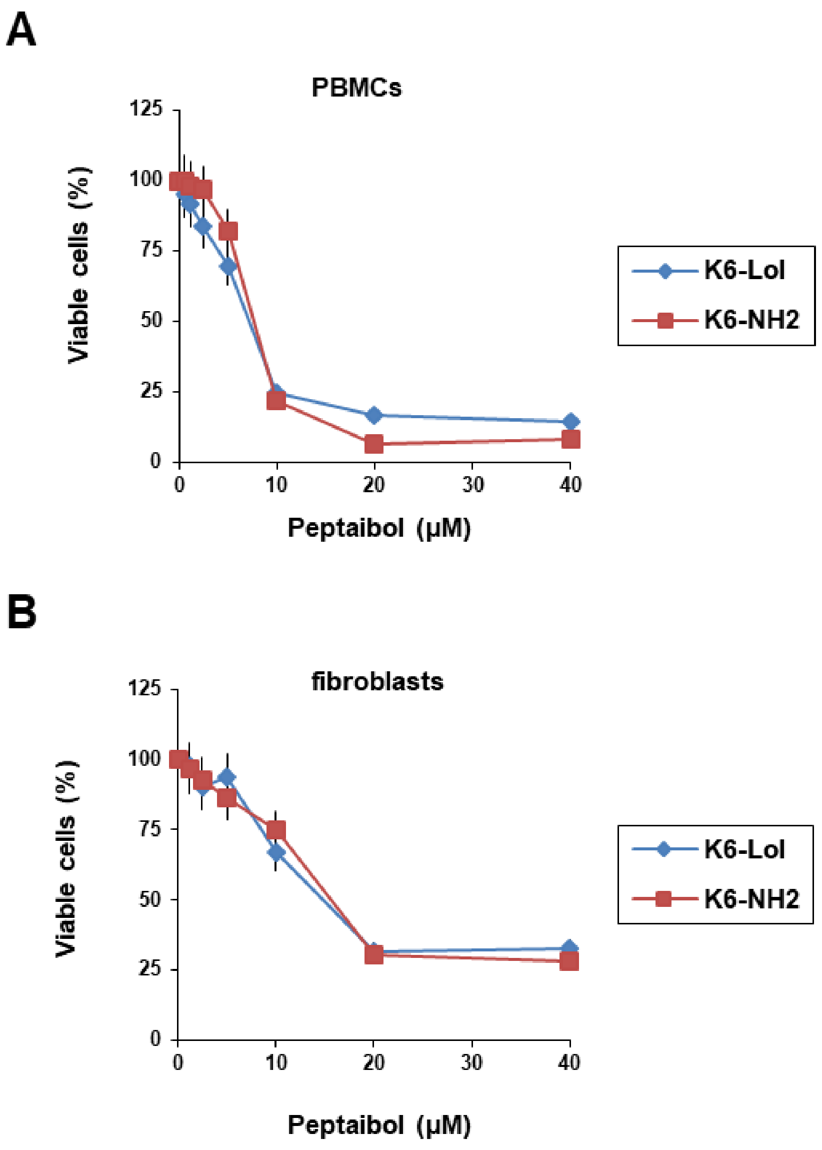

Supplemental Figure S7. Cytotoxic activiy of peptaibols in human PBMCs and fibroblasts. PBMCs and fibroblasts were cultured with peptaibols. After 24 hours (**A**) cell viability was evaluated by MTS assay for PBMCs (**B**) and by MTT assay for adherent fibroblasts. Results are mean and SD of three independent experiments.

Supplemental Figure S8

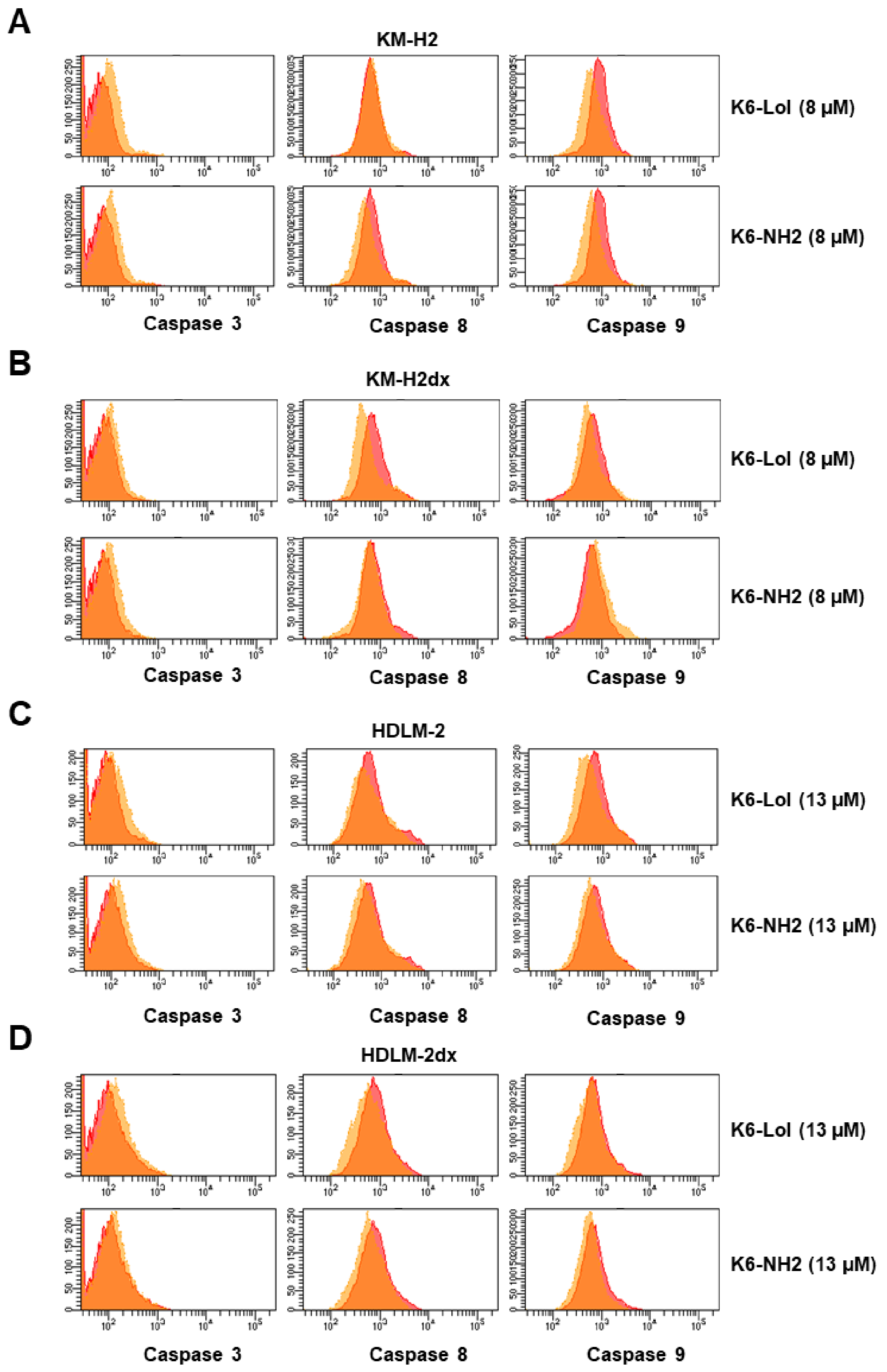

Supplemental Figure S8. Caspases activation by peptaibols in HRS cells. HRS cells were incubated with peptaibols (yellow histograms). After 24 hours Caspase 3, 8 and 9 activation was evaluated using fluorochrome-labeled inhibitors of caspases (FLICA) of the CaspaTag Caspase 3, 8 and 9 In Situ Assay Kit, Fluorescein (Millipore) and evaluated by flow cytometry.

Supplemental Figure S9

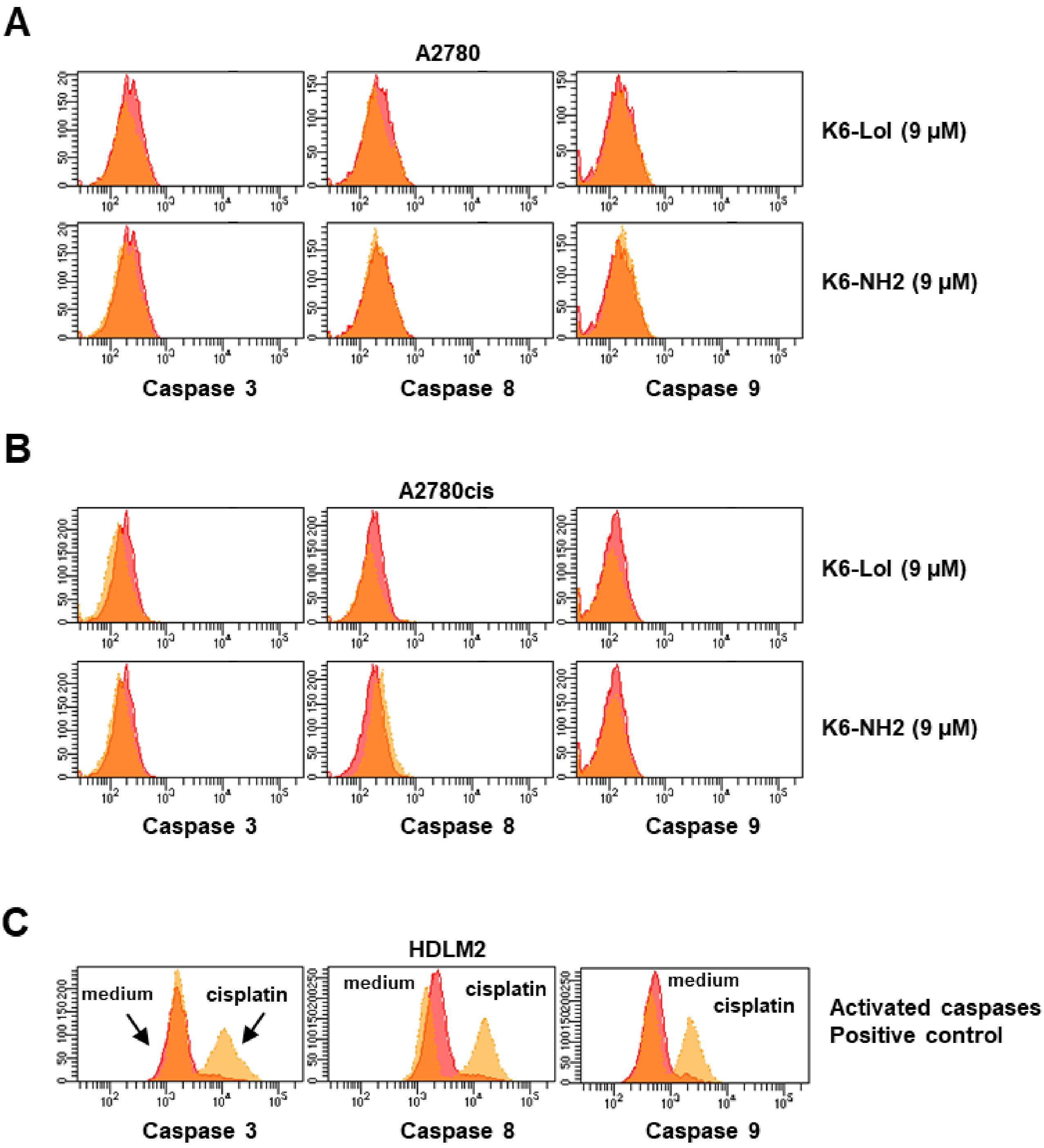

Supplemental Figure S9. Caspases activation by peptaibols in OvCa cells and by cisplatin in HDLM-2 cells. (**A, B**) OvCa cells were incubated with peptaibols (yellow histograms), and (**C**) HDLM2 cells were incubated with cisplatin (10 µM) and used as positive control for caspase activity. After 24 hours Caspase 3, 8 and 9 activation was evaluated using fluorochrome-labeled inhibitors of caspases (FLICA) of the CaspaTag Caspase 3, 8 and 9 In Situ Assay Kit, Fluorescein (Millipore) and evaluated by flow cytometry

Supplemental Figure S10

— **K6-NH2** in the presence of L428 cancer cell lines
— **control** L428 cancer cell lines before treatment with the peptide

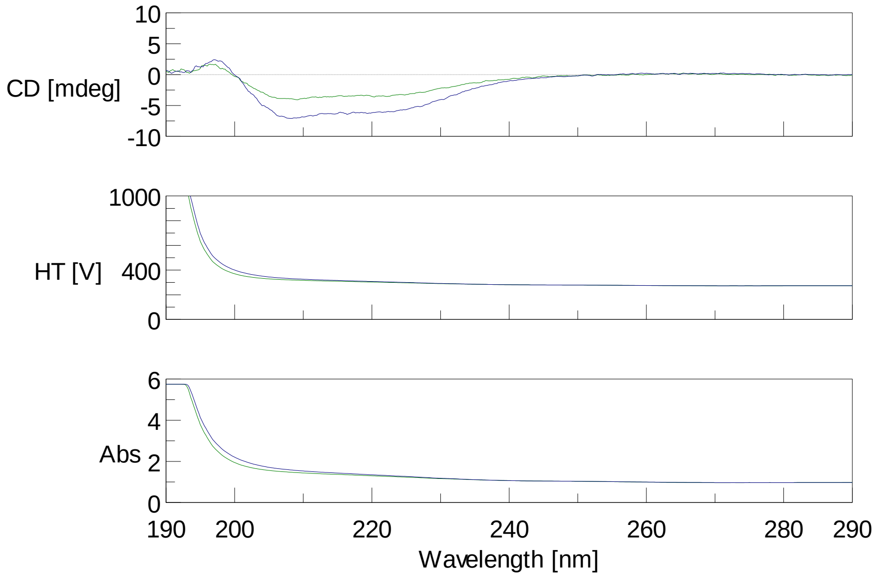

— **K6-Lol** in the presence of L428 cancer cell lines
— **control** L428 cancer cell lines before treatment with the peptide

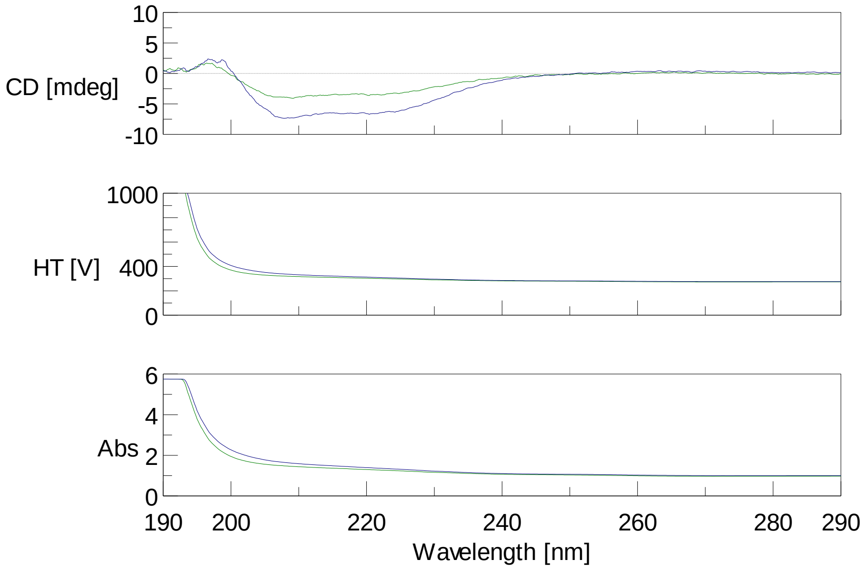

Supplemental Figure S10. Overlap of the original signal recorded for peptides K6-NH2 (top panel) and K6-Lol (bottom panel) in the presence of cancer cells (blue line) onto the background signal (green line). The background signal is the control CD spectrum of the cells before treatment with the peptide. As it can be seen, the array of chiral molecules in cells create a significant background signal, nonetheless the signal of the peptide is clearly visible.

Supplemental Figure S11: K6-Lol Characterizations

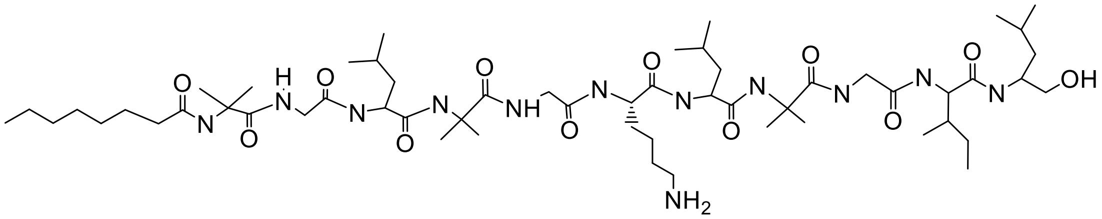

A HPLC profile

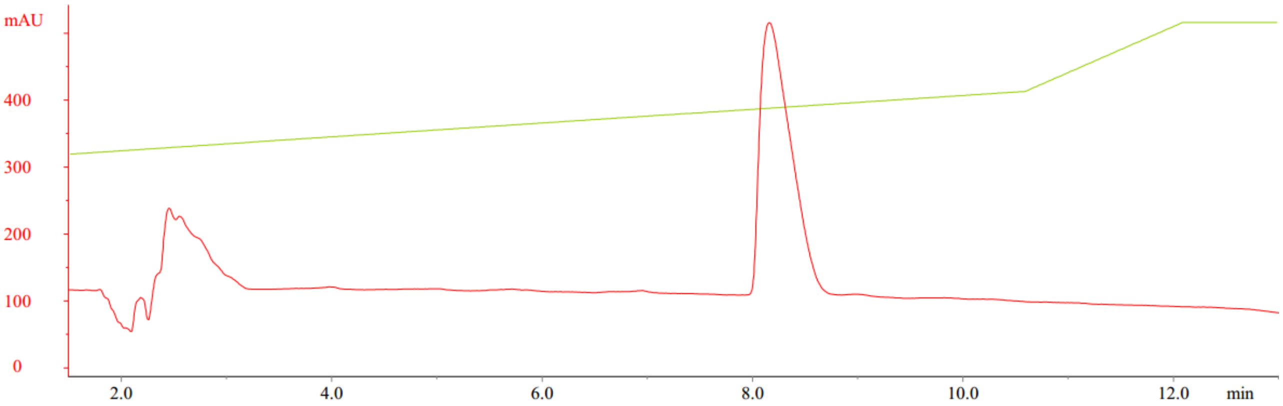

Supplemental Figure S11(**A**). HPLC profile of K6-Lol. Experimental conditions: 60- 80%B in 10 minutes; flux: 1mL/min; column: Agilent Zorbax RX-C_{18}. Retention time R_t = 8.16 min. Purity: 99%.

B High Resolution (HR)-ESI Mass Spectrometry (MS) spectrum

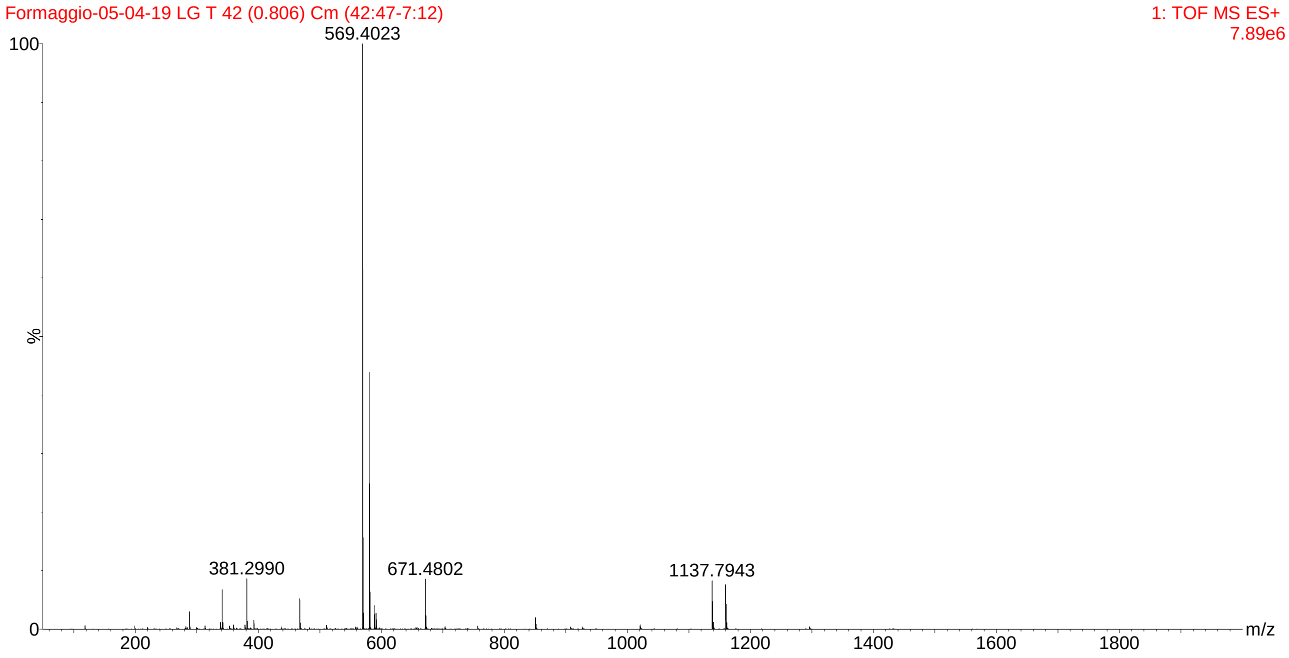

Supplemental Figure S11(**B**). HR-ESI MS spectrum of K6-Lol. $[M+H]^+_{calcd.}$ = 1137.7940 m/z. $[M+H]^+$ = 1137.7943 m/z; $[M+Na]^+$ = 1159.78 m/z; $[M+2H]^{++}$ = 569.40 m/z; $[M+H+Na]^{++}$ = 580.39 m/z. The peaks at m/z = 341.3078, 381.2990 and 671.4802 come from the instrument and couldn't be removed.

Supplemental Figure S12 . K6-Lol Characterizations

^{1}H NMR spectrum

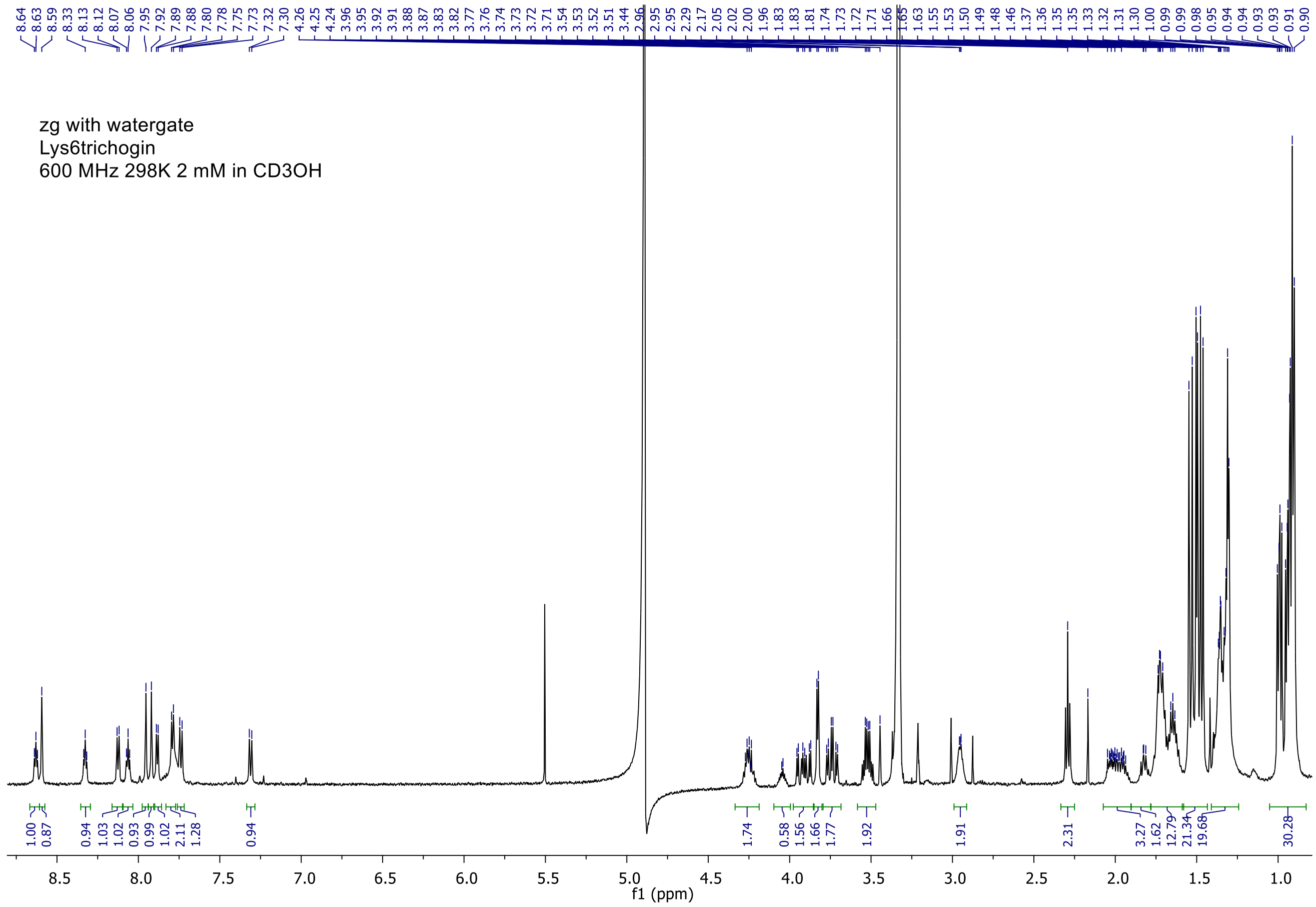

Supplemental Figure S12 . K6-Lol Characterizations

^{1}H-NMR spectrum of K6-Lol. Experimental conditions: 600 MHz, 298 K, peptide concentration: 2mM, solvent: CD_3OH.

Supplemental Figure S13: K6-NH2 Characterizations

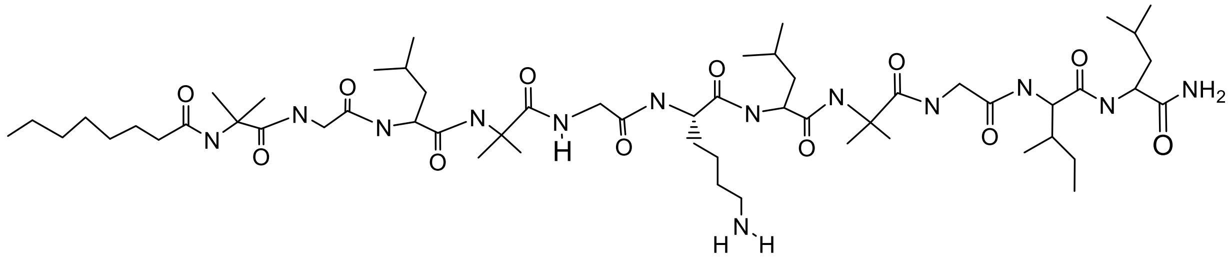

Chemical Formula: $C_{56}H_{103}N_{13}O_{12}$
Exact Mass: 1149,78

A HPLC profile

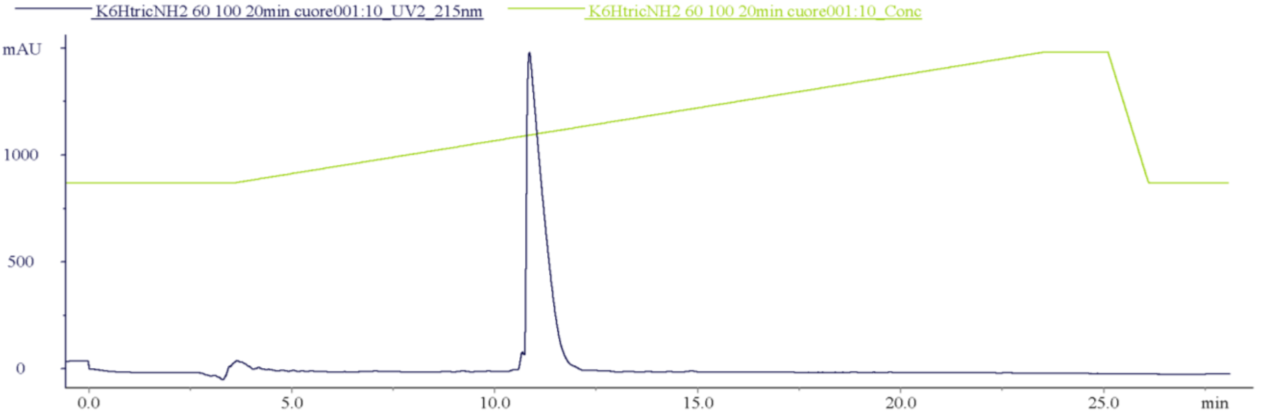

Supplemental Figure S13(**A**). HPLC profile of K6-NH2. Experimental conditions: 60- 100%B in 20 minutes; flux: 1mL/min; column: Agilent Zorbax RX-C_{18}. R_t = 10.85 min. Purity: 98%.

B HR-ESI MS spectrum

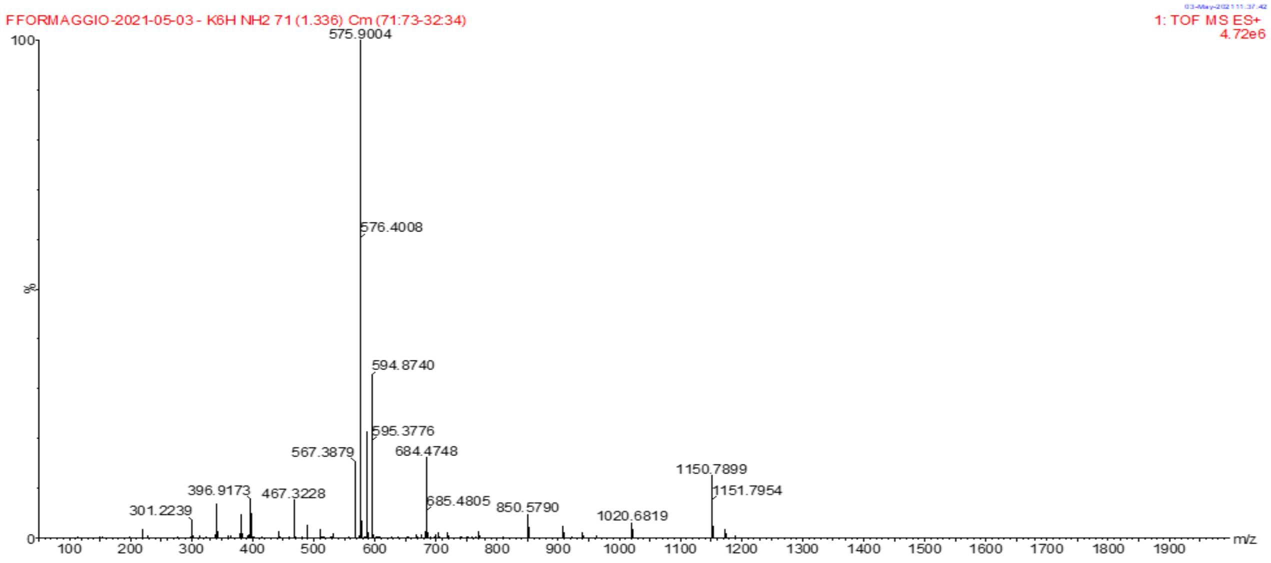

Supplemental Figure S13(**B**). HR-MS spectrum of K6-NH2. $[M+H]^+_{calcd.}$ = 1150.7849 m/z. $[M+H]^+$ = 1150.7899 m/z; $[M+Na]^+$ = 1172.7688 m/z; $[M+2H]^{++}$ = 575.9004 m/z; $[M+H+Na]^{++}$ = 586.8901 m/z; $[M+H+K]^{++}$ = 594.8740 m/z. The peaks at m/z = 341.3078, 381.2990 and 671.4802 come from the instrument and couldn't be removed.

Supplemental Figure S14: K6-NH2 Characterizations

^{1}H NMR spectrum

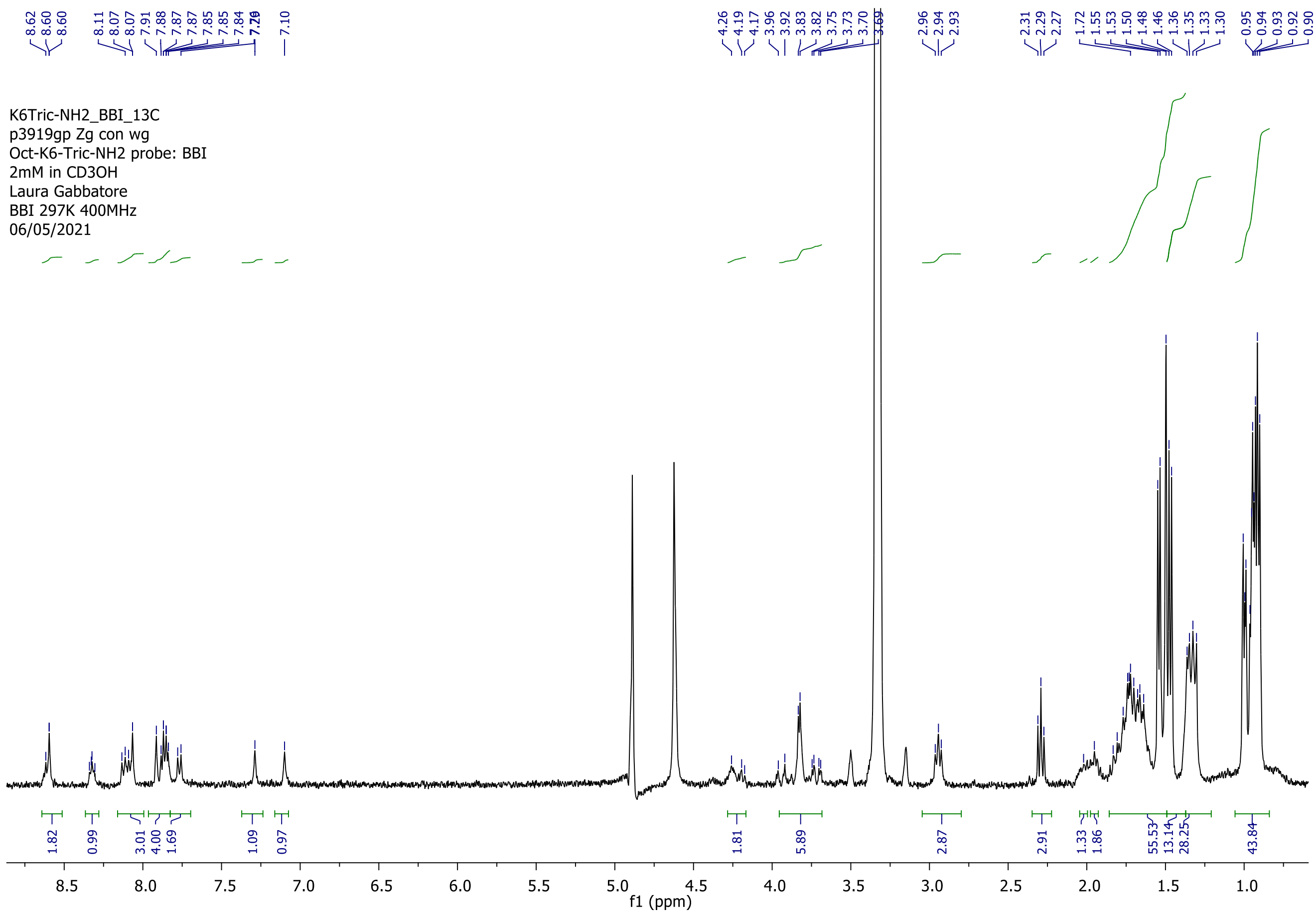

Supplemental Figure S14. ^{1}H-NMR spectrum of K6-NH2. Experimental conditions: 600 MHz, 297 K, peptide concentration: 2mM, solvent: CD_3OH.

^{13}C NMR chemical shifts of K6-NH_2(101 MHz, CD_3OH) δ 177.10, 174.96, 174.27, 172.55, 171.12, 166.95, 156.50, 136.70, 128.87, 127.40, 126.07, 64.64, 59.47, 56.57, 56.31, 56.01, 54.97, 53.96, 39.80, 39.42, 39.31, 39.16, 36.27, 35.43, 31.66, 30.06, 29.92, 29.34, 29.02, 28.81, 26.85, 26.78, 26.54, 26.47, 25.84, 25.35, 25.07, 25.03, 24.59, 24.28, 23.17, 22.76, 22.64, 22.28, 21.80, 21.43, 21.25, 20.63, 20.25, 14.59, 10.50.

Supplemental Figure S15: K6-FITC Characterizations

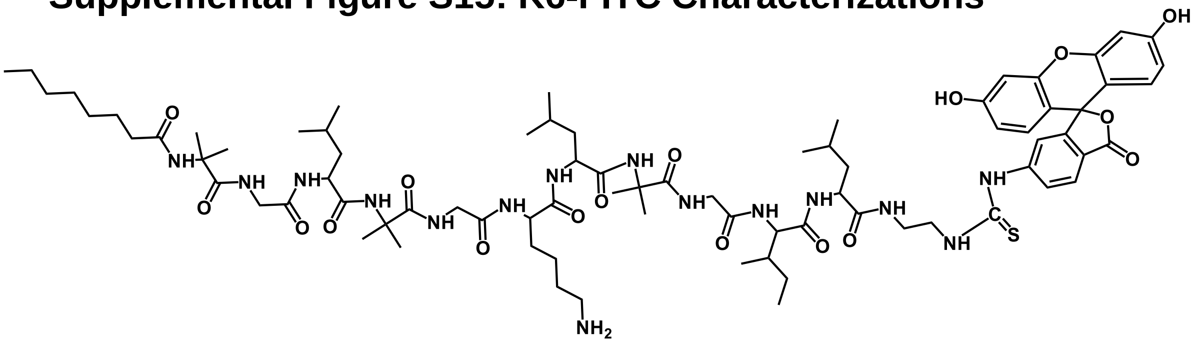

Chemical Formula: $C_{79}H_{119}N_{15}O_{17}S$
Exact Mass: 1581,86

A HPLC profile

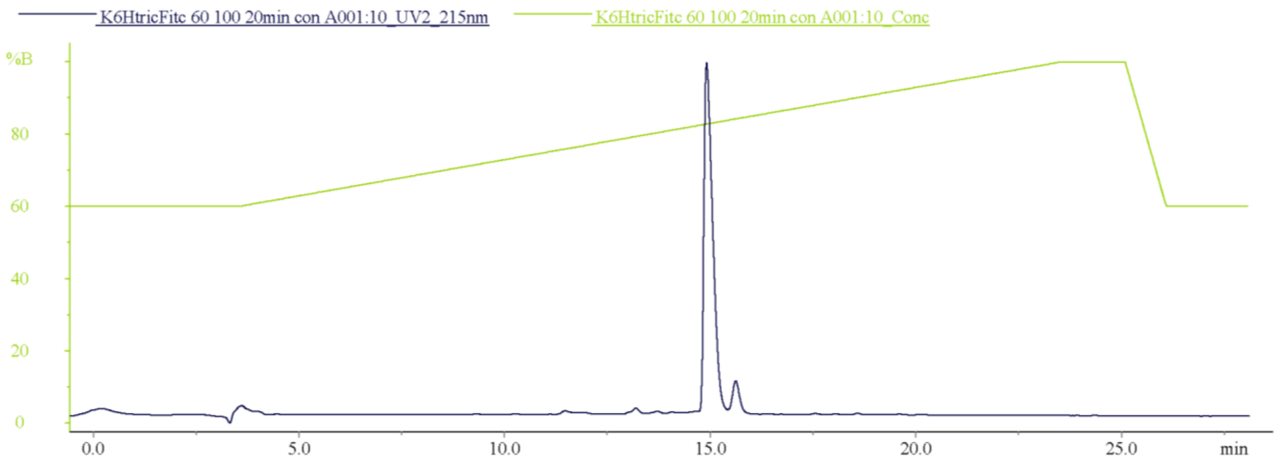

Supplemental Figure S13(**A**) HPLC profile of K6-FITC. Experimental conditions: 60- 100%B in 20 minutes; flux: 1mL/min; column: Agilent Zorbax RX-C_{18}. R_t = 14.91 min. Purity: 96%.

B HR-ESI MS spectrum

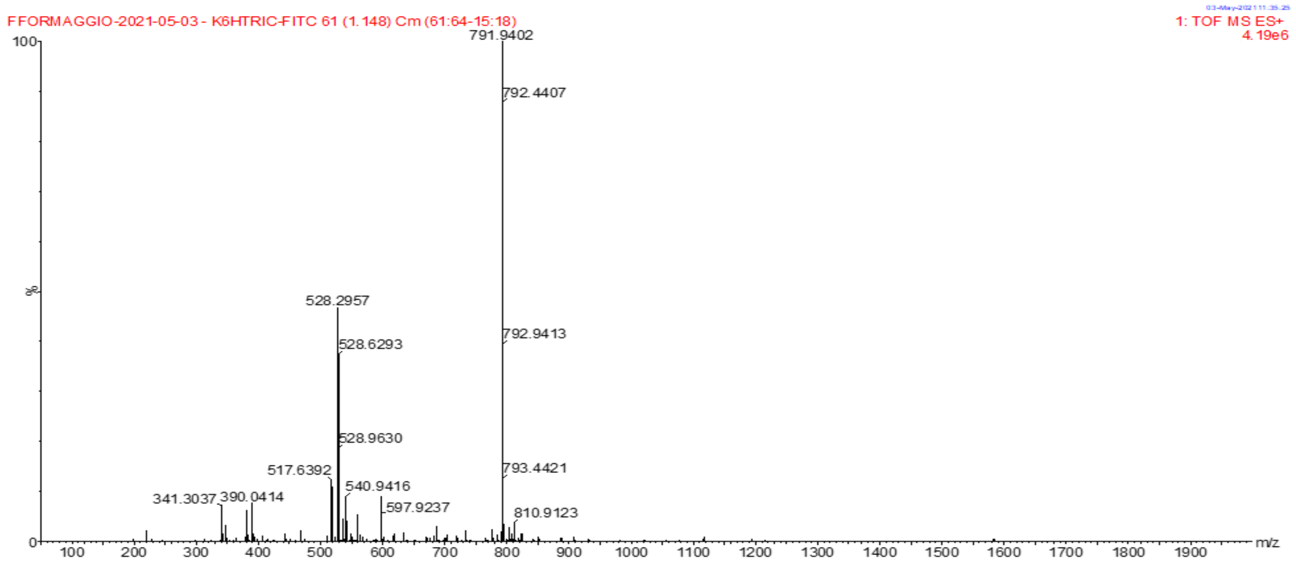

Supplemental Figure S15 HR-ESIMS spectrum of K6-FITC. $[M+2H]^{2+}_{calcd.}$ = 791,9385 m/z. $[M+2H]^{2+}$ = 791.9402 m/z; $[M+3H]^{3+}$ = 528.2957 m/z. The peaks at m/z = 341.3078, 381.2990 and 671.4802 come from the instrument and couldn't be removed.

Supplemental Figure S16: K6-FITC Characterizations

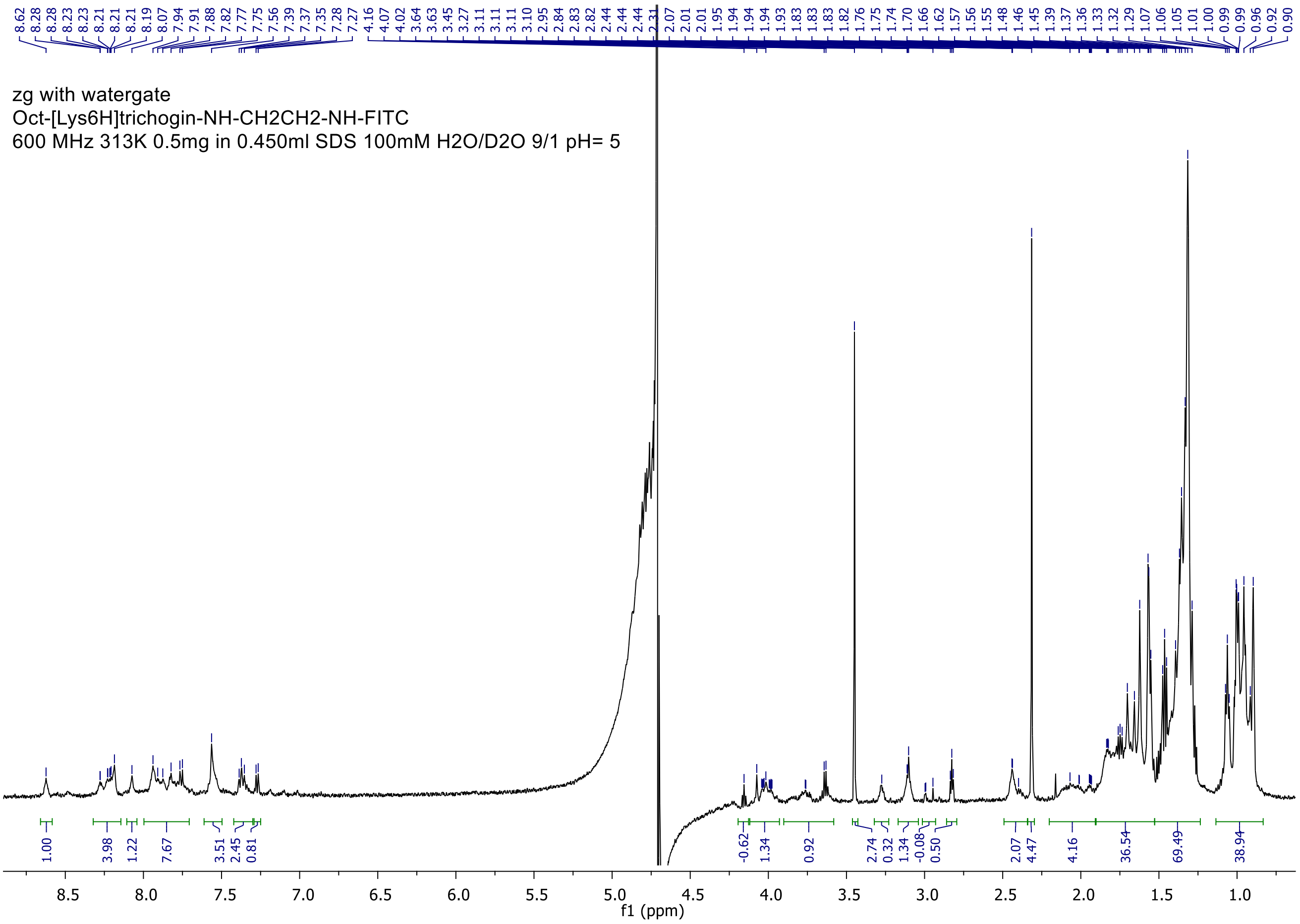

Supplemental Figure S16. ^{1}H-NMR spectrum of K6-FITC. Experimental conditions: 600 MHz 313 K, peptide concentration: 0.7mM, solvent: sodium dodecyl solfate (SDS)-d_{12} 100 mM in H_2O/D_2O 9/1 pH= 5.

Supplemental Figure S17: K6-FITC Characterizations

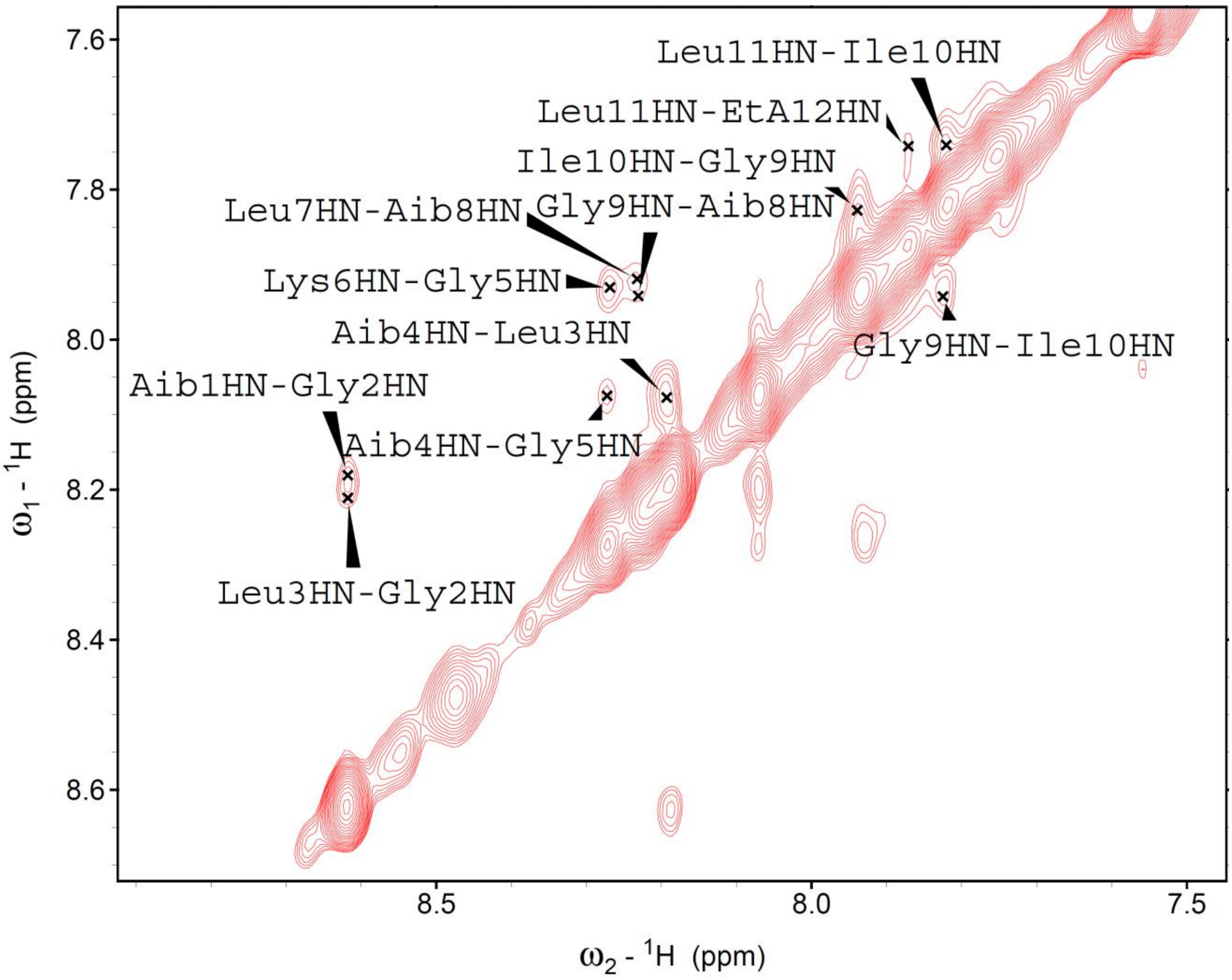

Supplemental Figure S17. Amide proton region of the 2D NMR ROESY spectrum of K6-FITC. Experimental conditions: 400 MHz, 298K, peptide concentration: 2 mM. Solvent: SDS-d_{12} 100 mM in H_2O/D_2O 9/1 pH= 5.
